# Knowledge-based mechanistic modeling accurately predicts disease progression with gefitinib in *EGFR*-mutant lung adenocarcinoma

**DOI:** 10.1038/s41540-023-00292-7

**Published:** 2023-07-31

**Authors:** Adèle L’Hostis, Jean-Louis Palgen, Angélique Perrillat-Mercerot, Emmanuel Peyronnet, Evgueni Jacob, James Bosley, Michaël Duruisseaux, Raphaël Toueg, Lucile Lefèvre, Riad Kahoul, Nicoletta Ceres, Claudio Monteiro

**Affiliations:** 1 Novadiscovery SA, Pl. Giovanni da Verrazzano, Lyon, 69009 Rhône France; 2 Respiratory Department and Early Phase, Louis Pradel Hospital, Hospices Civils de Lyon Cancer Institute, Lyon, 69100 France; 3 Cancer Research Center of Lyon, UMR INSERM 1052 CNRS 5286, Lyon, France; 4 Université Claude Bernard Lyon 1, Université de Lyon, Lyon, France; 5 Janssen-Cilag, France, 1, rue Camille Desmoulins - TSA 60009, Issy-Les-Moulineaux Cedex 9, Issy-Les-Moulineaux, 92787 France

**Keywords:** Differential equations, Cancer, Cancer

## Abstract

Lung adenocarcinoma (LUAD) is associated with a low survival rate at advanced stages. Although the development of targeted therapies has improved outcomes in LUAD patients with identified and specific genetic alterations, such as activating mutations on the epidermal growth factor receptor gene (*EGFR*), the emergence of tumor resistance eventually occurs in all patients and this is driving the development of new therapies. In this paper, we present the In Silico *EGFR*-mutant LUAD (ISELA) model that links LUAD patients’ individual characteristics, including tumor genetic heterogeneity, to tumor size evolution and tumor progression over time under first generation EGFR tyrosine kinase inhibitor gefitinib. This translational mechanistic model gathers extensive knowledge on LUAD and was calibrated on multiple scales, including in vitro, human tumor xenograft mouse and human, reproducing more than 90% of the experimental data identified. Moreover, with 98.5% coverage and 99.4% negative logrank tests, the model accurately reproduced the time to progression from the Lux-Lung 7 clinical trial, which was unused in calibration, thus supporting the model high predictive value. This knowledge-based mechanistic model could be a valuable tool in the development of new therapies targeting *EGFR*-mutant LUAD as a foundation for the generation of synthetic control arms.

## Introduction

Lung cancer is one of the most frequently diagnosed cancers and the leading cause of cancer mortality worldwide^1,2^. More than 40% of newly diagnosed lung cancers are in a metastatic state^3^. Based on European and American guidelines (respectively^4,5^), the main treatment options currently available for patients with lung adenocarcinoma (LUAD)—representing 40% of all lung cancer^6^, are surgery, radiation therapy, chemotherapy, immunotherapy, and targeted therapy.

Alterations such as gene mutations or fusion lead to uncontrolled receptor tyrosine kinases (RTK) signaling and an oncogenic signal, leading to strong activation of downstream pathways converging on common signaling effectors that elicit tumor development^7^. Molecularly targeted therapies have markedly improved clinical outcomes in patients with LUAD defined by the detection of oncogenic mutation or fusion in RTK-like epidermal growth factor receptor (EGFR). The EGFR tyrosine kinase inhibitor (TKI) gefitinib was the first targeted therapy for the treatment of advanced EGFR-mutant LUAD approved by both the European Medical Agency (EMA) and the Food & Drug Administration^8^. However, as all *EGFR*-mutant LUAD eventually develop resistance to treatment this disease is still one of the most deadly cancers^9^.

The development of new molecularly targeted therapies comes with a high human, time, and financial cost^10–12^. Yet, a large amount of data and knowledge resulting from biological experiments of last decades at different scales (from the molecular level to the population level) and in various conditions (in vitro cultivated cells, animal experiments, human studies) are now publicly available for integration to support new insights and progress^13^. Drug development decision making could benefit from being informed and rationalized by the integration of these heterogeneous data. Knowledge-based mechanistic computational models represent a valuable tool to bridge quantitatively experimental data that are heterogeneous in scale and nature. In particular, they provide insights to study the rare mutation combinations, such as *KRAS* (Kirsten rat sarcoma gene) or *BRAF* (B rapidly accelerated fibrosarcoma gene) co-occurring with *EGFR* mutation. They can provide both the dynamics of the biological entities included in the modeling and clinical outputs of patients. Another interest of mechanistic disease models lies in their modularity: models of other treatments can therefore be easily integrated into such models of physiopathological processes^14^.

Such a model can be used to predict the clinical outcomes of a virtual patient, implemented as a digital twin of a real world patient, in response to distinct sets of treatments, allowing prediction of clinical outcomes of a target population of such patients, based on the corresponding virtual population (Box 1) and can serve as a support for clinical development of new drugs.

Such mechanistic models have been developed in the past for oncology application. For instance, Milberg et al.^15^ developed a model of detailed anti-tumor immune response in a context of melanoma, including several immune cell interactions linked to tumor diameter evolution. Dogra et al.^16^ reported a model linking the pharmacokinetics of several treatments on cell cycle progression in triple-negative breast cancer. Others, such as Barber et al.^17^ or Yu et al.^18^ used statistical models to link tumor characteristics with the clinical outcome progression-free survival. In 2020, Nagase et al.^19^ published a Bayesian model tumor radius evolution of EGFR-mutant NSCLC treated with 1st generation TKI. However, to our knowledge, a mechanistic model that targets the same population, and that links key molecular and cellular cancer evolution actors to disease progression and clinical outcomes, as observed in clinics, is still missing.

Based on the recommendations from EMA^20^ with respect to physiologically based pharmacokinetic modeling and the American Society of Mechanical Engineers (ASME) Verification & Validation (V&V) 40 standard published by the FDA, model development could be summarized in four main steps: (1) definition of model context of use^20,21^, (2) construction of a knowledge model describing the patho-physiological interplay of biological phenomena within the context of use, (3) implementation of a computational model by translating knowledge model into mathematical equations, (4) calibration of the model parameters to ensure that simulations reproduce expected behaviors observed in the real world.

In addition to the four main steps, we propose a fifth one (5), namely the validation of the model^22^, in order to assess model credibility by challenging its predictability in reproducing real-world data that were not used to build the model nor to calibrate it.

We present in this article a mechanistic model built based on those guidelines with the additional validation step (fifth step), integrating multiscale phenomena, with a context of use to predict tumor evolution and disease progression over time of *EGFR*-mutant LUAD patients treated with gefitinib. Other settings such as additional treatments or placebo in humans are deemed out of the scope of this work. We present here the in silico strategy used to build the In Silico *EGFR*-mutant LUAD (ISELA) model, its validation ensuring the reliability of its prediction and the use of the model to identify individual characteristics linked to clinical outcome.

Box 1 Definition of virtual patients and virtual population A Virtual Population is a modeling technique used to describe a cohort of virtual patients. Each individual virtual patient is characterized by a unique set of parameter values, which are named descriptors. The number of patients is specified. The vector of descriptor values are sampled from a vector of patient descriptor distributions (e.g., age, sex, or co-mutation profile), using their probabilistic distributions and correlations derived from the target population, in order to represent its reported variability. With these inputs to the computational model, the individual outcomes of the virtual patients (i.e., tumor size evolution and time to progression for the In Silico *EGFR* mutant LUAD (ISELA) model) are simulated.

## Results

### Visual predictive checks

**Fig. 1:**
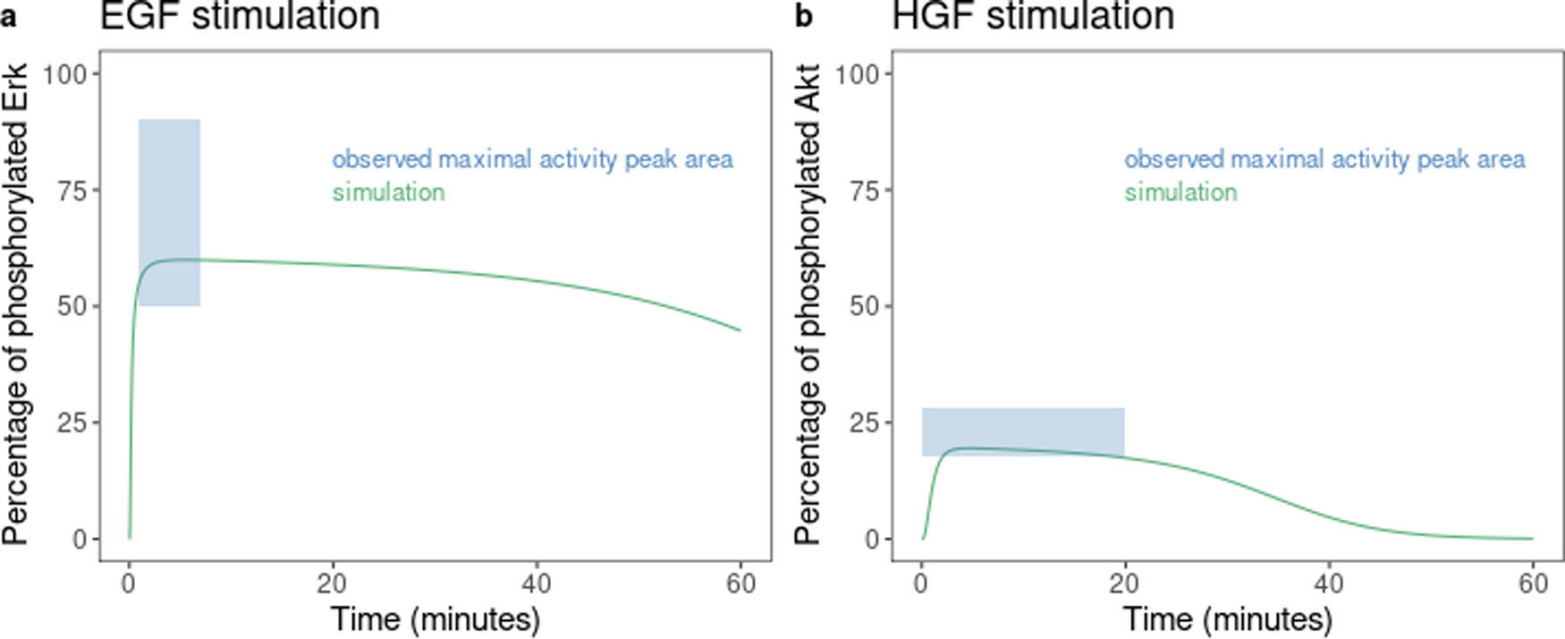
Quantitative visual predictive check of calibration step 1 results. The correspondence between simulation outcome and observed data, after calibration, was assessed (**a**, **b**) for the activation of pathways downstream to EGFR and cMET (cellular mesenchymal epithelial transition). The observational data corresponds to the area defined by both maximal phosphorylation rate (dashed line) and time of that maximum following the indicated stimulation.

**Fig. 2:**
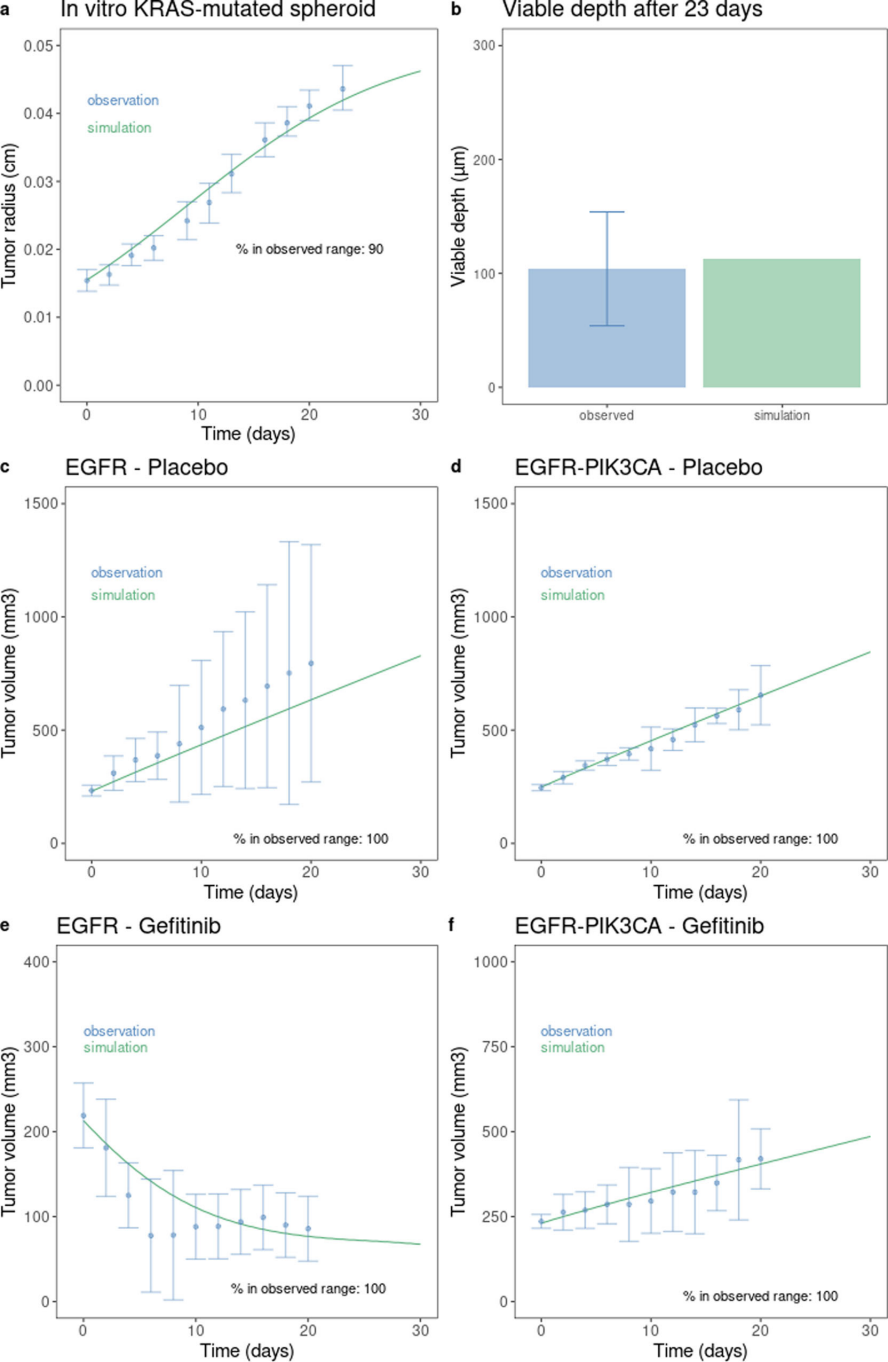
Quantitative visual predictive check of calibration step 2 results. The correspondence between simulation outcome and observed data, after calibration, was assessed for tumor evolution. **a** Evolution of spheroid tumor size; the observational data corresponds to the mean tumor radius overtime with estimated error bars (taken as maximum between 2 standard deviations and 20% of the mean). **b** Maximal depth at which living tumor cells can be found (viable depth) in tumor spheroids; the observational data corresponds to estimated viable depth observed on in vitro spheroids. **c**–**f** The observational data corresponds to the mean tumor volume overtime, with estimated error bars (taken as maximum between 2 standard deviations and 20% of the mean). For the evolution of tumor radius and volume, the percentage of data intervals that are reached by the model simulation is also indicated.

**Fig. 3:**
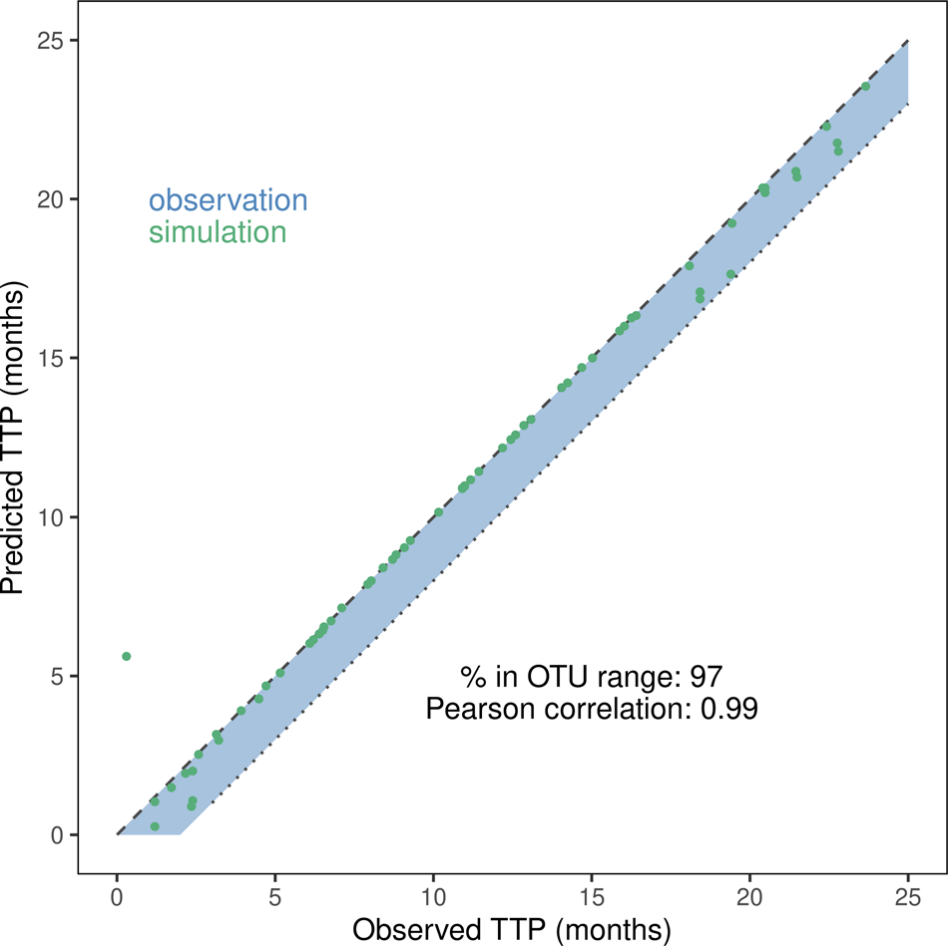
Visual predictive check of the calibration step 3 results. Predicted versus observed time to progression (TTP) are displayed for the virtual patients (single dot) best matching each of the real world patients. Each prediction (dot) is considered accurate if the associated point lies in the blue ribbon representing the interval between TTP (dashed line) and TTP minus 2 months (dotted line), 2 months being the time lapse between two medical visits as reported in ref. ^46^, namely the observation time uncertainty^22^. In brief, when medical visits occur every n month, then if TTP is reported at visit m, it means that the exact time of progression occurred between the visit m-1 and the visit m, hence the uncertainty in TTP measurement. The percentage of prediction within the observed time uncertainty range (% in OTU) corresponds to the percentage of dots that are in the uncertainty area (blue area, reported TTP - 2 months). Pearson correlation coefficient is also provided.

As a verification criterion of calibration success, as well as correct estimation of parameter values and distribution amongst the population, visual predictive checks were performed on the experimental dataset used for calibration (Figs. 1, 2 and 3).

For the in vitro calibration of the model, the model faithfully reproduced the time at which ERK and AKT proteins reach their maximal activation levels, and the maximal activation observed (following epidermal growth factor (EGF) or hepatocyte growth factor (HGF) stimulation) as shown in Fig. 1.

On in vitro *KRAS* mutated spheroid simulation, the model output matched the experimental data, both in terms of tumor radius evolution, and maximal depth for cell viability observed in these conditions (Fig. 2a, b). On the mice xenografted with patient-derived tumor (carrying exon 19 deletion mutation, with or without co-occurrence of *PIK3CA* mutation), either treated with gefitinib or untreated and based on the dynamics observed in literature, the ISELA model reproduces accurately the evolution of tumor volume over time (Fig. 2c–f).

The selected range of acceptable model output variation, materialized by the error bars, was defined as the maximum between two times the associated standard deviations and 20% of the mean. This approach allows coverage of heterogeneous calibration datasets: we were able to constrain the model on datasets composed of one or few numbers of experiments and/or datapoints, as well as datasets lacking standard deviation. Finally, the penalizations were applied to the selected range of variation without assigning specific weight to the mean. To note, in one condition (namely EGFR mutant with placebo), the simulation tended to provide a tumor volume that was slightly lower than the observed one, while remaining in the experimental uncertainty that was huge in this particular setting.

In the human setting, the ISELA model was able to match a realistic TTP for 97% patients included in the calibration process (one virtual patient displaying a higher TTP than their real world counterpart), supporting its reliability with respect to clinical outcome prediction (Fig. 3).

To conclude, calibration constrained the ISELA model by finding a set of parameter values allowing it to represent biological behaviors consistent with data extracted from the literature. Thus these steps increased the credibility of the ISELA model. However, a validation step is needed to formally assess the performance of the model and in particular its prediction capacity in its context of use.

### Validation process

We performed a validation process to assess the reliability of the ISELA model predictions. We aimed to ensure that the model is able to reproduce biological and clinical behaviors extracted from independent clinical datasets that were not used to build nor calibrate the model. In the following, we compare the ISELA model predictions with the data extracted from ref. ^23^. The model is deemed as successfully validated if it respects the thresholds set in the “Materials and methods” section on raw coverage and bootstrapped LR-test thresholds.

#### Generation of the virtual population

As explained in the “Materials and methods” section, we generated a virtual population ten times larger than the real population size. Table 1 provides statistical comparison illustrating how close the generated virtual population is from the clinical data, as detailed in “Materials and methods” section.

**Table 1:** Comparison of baseline characteristics between virtual population and real population from ref. ^23^.

Characteristics	Lux-Lung 7 (*n* = 159)	Virtual population (*n* = 1190^a^)	*p*-value
**Gender,** ***n*** **(%)**			
Men	53 (33.3)	100 (33.6)	1.0
Women	106 (66.7)	790 (66.4)	
Age in years - median (range)	63 (36-89)	62.7 (32.7–88.7)	0.9
**EGFR mutation,** ***n*** **(%)**			
Exon 19 deletions	93 (58.5)	701 (58.9)	0.8
Exon 21 L858R point mutation	66 (41.5)	489 (41.1)	
**Smoking status,** ***n*** **(%)**			
Never smoker	106 (66.7)	789 (66.3)	0.98
Former smoker	19 (11.9)	136 (11.4)	
Current smoker	34 (21.4)	265 (22.3)	
**Ethnicity,** ***n*** **(%)**			
Asian (%)	88 (55)	712 (55.2)	0.93
Non-asian (%)	71 (45)	588 (44.8)	
**Clinical stage at screening,** ***n*** **(%)**			
IIIb	3 (1.9)	33 (2.8)	0.73
IV	156 (98.1)	1157 (97.2)	

Statistical comparisons were made using the Fisher test, except for age which was assessed with a t-test.

^a^The virtual population was built with 1190 virtual patients, which corresponds to 10 times the size of the real population that displayed a progression event (TTP), as detailed in the “Materials and methods” section.

The Virtual population did not differ significantly from the real population, for any of the compared characteristics: the virtual population generated was therefore representative of the provided Lux-Lung 7 population characteristics. As a result, simulation outputs can rightfully be compared to real world clinical data, as the inputs match.

We then compared the outputs of the simulations to the Lux-Lung 7 inferred TTP to evaluate whether the ISELA model reproduces accurately the Lux-Lung 7 trial.

#### Comparison of survival curves between simulated and real population

**Fig. 4:**
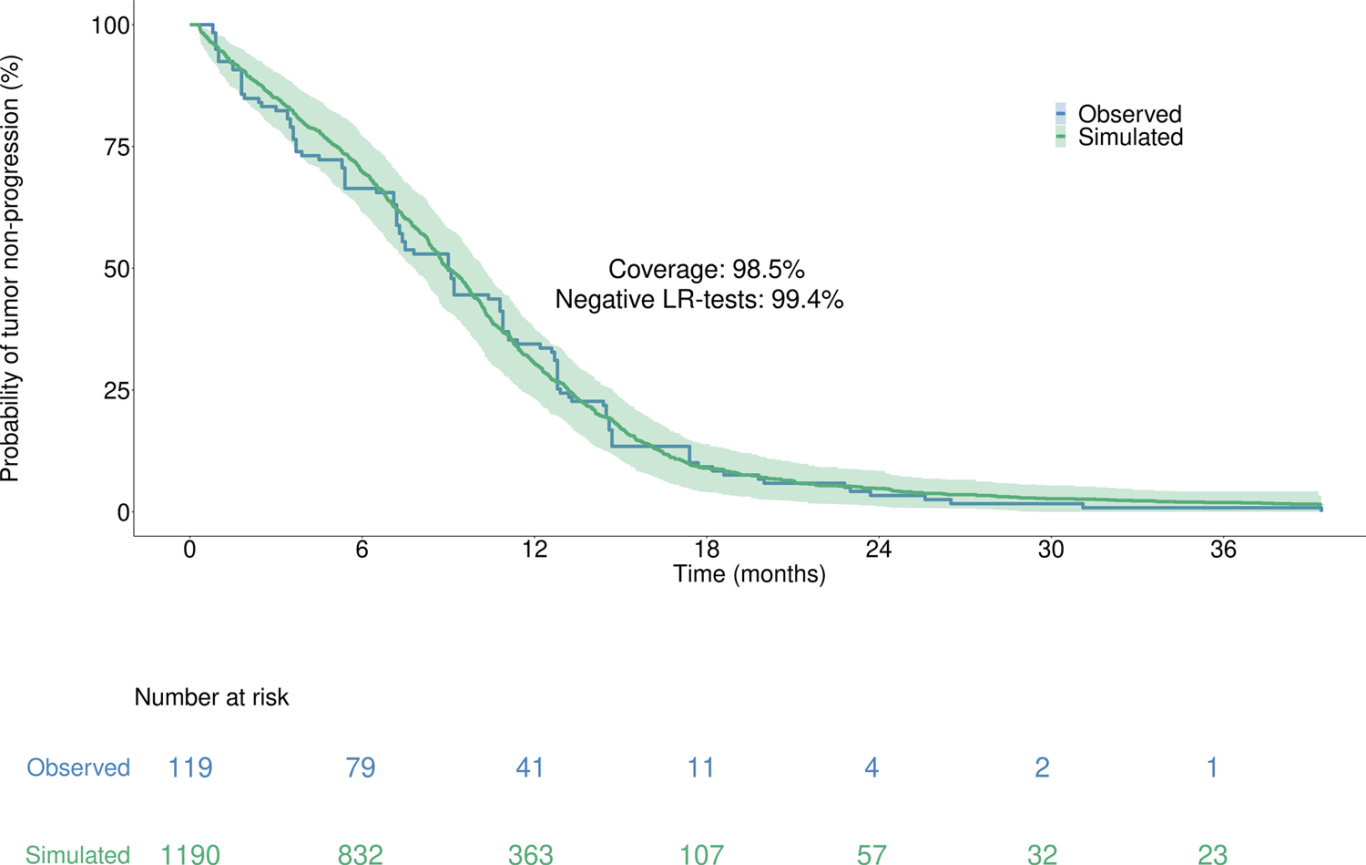
Kaplan–Meier curves illustrating the TTP for the observed population of the Paz-Arés et al. dataset and the corresponding simulated Virtual Population. The raw time-to-event curve from literature (blue curve) represents TTP deduced from Paz-Ares et al. The simulated time-to-event curve (light green curve) is fitted with a prediction interval (PI) computed by bootstrapping (light green area). The validation metrics are displayed in the middle of the plot, and are detailed in the section “Virtual population generation and statistical analyses for validation”. The number of patients at risk is shown below the plot. LR log-rank, PI prediction interval. All patients received a daily dose of 250 mg gefitinib starting at day 0 of the simulation onwards.

We provided the output of the model using a classical Kaplan–Meier survival curve, defined by the with the survival curve computed on the entire Virtual Population and with its 95% bootstrapped prediction interval (PI), overlaid with the TTP deduced from the Kaplan–Meier curves extracted from ref. ^23^ (Fig. 4).

As seen in Fig. 4, the ISELA model fulfills both validation criteria detailed in the “Materials and methods” section: 98.5% of experimental data are covered by the model prediction interval and only 0.6% of bootstrapped LR tests are significant (not able to reject the null hypothesis defined as: no difference between observed and simulated populations). These results support that observed and simulated TTP data are not statistically different. The ISELA model is thus considered as validated, as per the initial objective. As a consequence it increases the credibility of both the model predictions, and its matching of the real world population.

#### Exploration of the individual tumor size evolution

Exploration of model outcomes within the virtual population of the validated ISELA model was performed on the tumor size evolution dynamics.

**Fig. 5:**
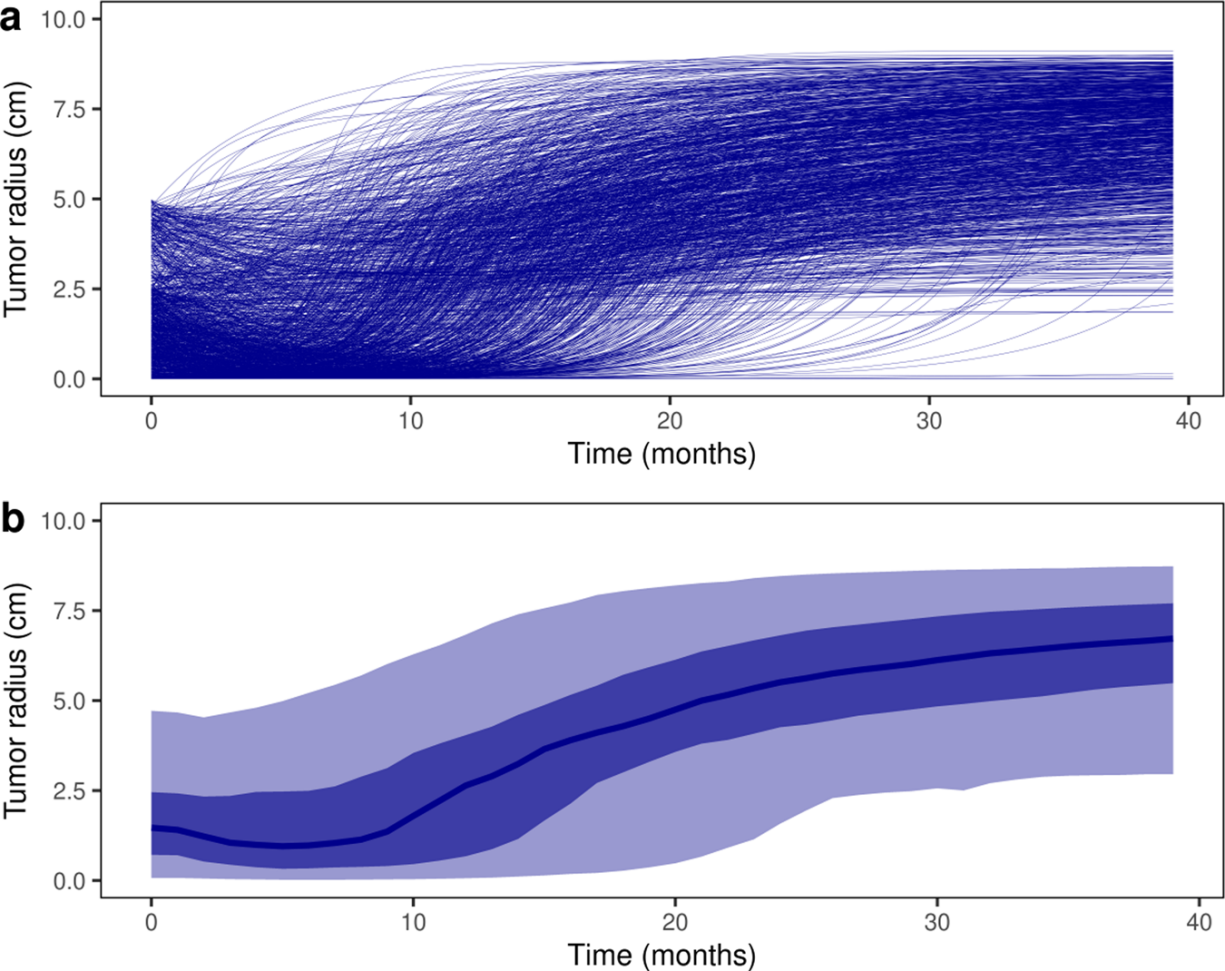
Individual and population tumor size evolution within the virtual population. **a** The individual tumor radius dynamics over time of each of the 1190 patients from the Virtual Population are represented. **b** The median (dark blue), 25–75% (intermediate blue), and 2.5–97.5% (light blue) confidence intervals are given for the overall population. **a**, **b** All patients received a daily dose of 250 mg gefitinib starting at day 0 of the simulation onwards.

The tumor radius evolution over time is an output of the ISELA model that was calibrated on in vitro and on mice with success (see Figs. 1 and 2). Tumor radius can be followed every day during the simulation for each individual patient treated with gefitinib, as displayed in Fig. 5. As expected, the vast majority of the patients show a decrease of tumor size at first, followed by a relapse of the tumor which becomes gefitinib-resistant and increases in size, though this relapse time differs among patients (see Fig. 5).

**Fig. 6:**
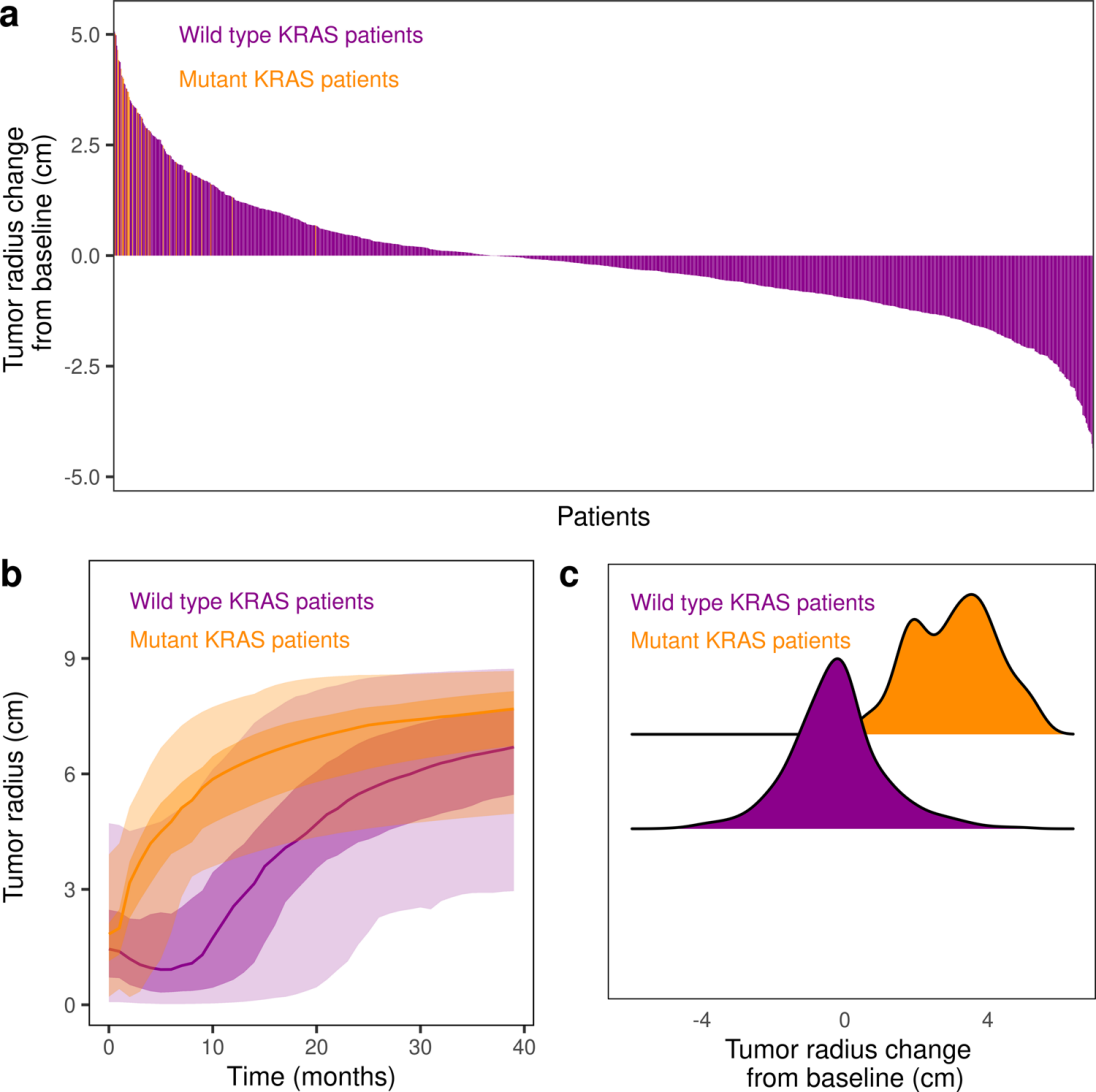
Evolution of tumor radius stratified by *KRAS* mutation. **a** The change in tumor radius at 6 months compared to baseline is displayed in a waterfall plot, with a stratification on *KRAS* mutation. **b** The median, 25–75%, and 2.5–97.5% confidence intervals are given for the two subpopulations carrying or not carrying the *KRAS* mutation. **c** The distribution of tumor radius change at 6 months compared to baseline is displayed for the two subpopulations carrying or not carrying the *KRAS* mutation. All patients received a daily dose of 250 mg gefitinib starting at day 0 of the simulation onwards.

When stratifying the patients based on the co-occurence of the *KRAS* mutation, it was noted that tumors harboring the *KRAS* mutation resist gefitinib, as expected^24^, compared to other tumors. Indeed, when size was compared at 6 months to baseline, different patterns were observed ranging from an increase in tumor size to a decrease in tumor size, patients harboring *KRAS* mutation being in the first case (Fig. 6).

**Fig. 7:**
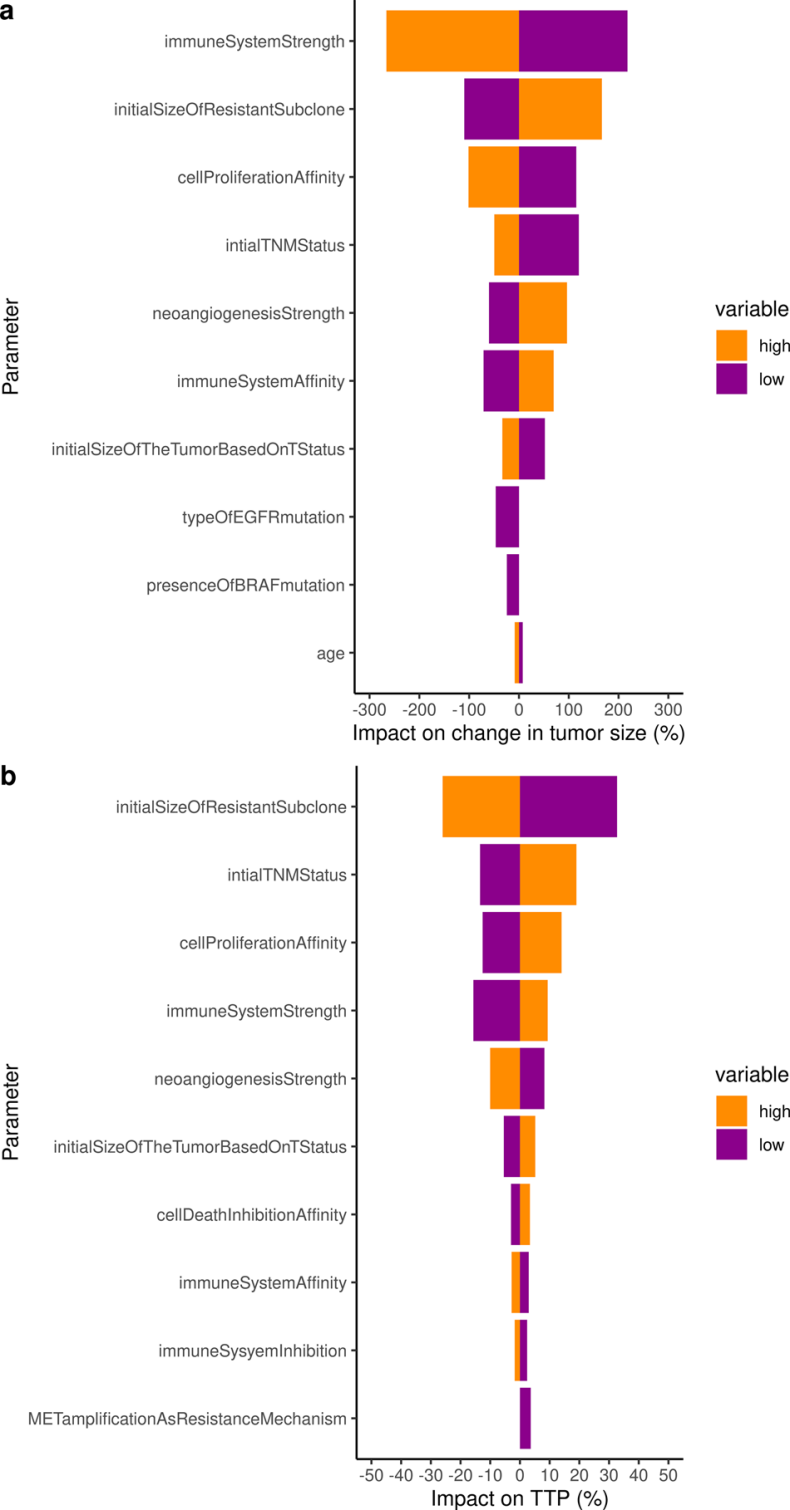
Sensitivity analysis of the ISELA model. A Virtual population of 5000 patients following the characteristics of the general population was generated and their tumor size (**a**) and TTP (**b**) analyzed by Tornado plots analysis to quantify the impact of each parameter on these outputs. The median change in tumor size was −14.3% and the median TTP was 8.06 months, values are provided as “100 × (value in the low/high category − median value)/ median value” and this represents the relative variability induced by each parameter, in percentage. The 10 most impactful parameters are provided in both cases, Note that version of the model, without clonal duplication, was used to estimate the impact of each and every parameter. This was done in order not to overwhelm the sensitivity analysis with 75 parameters that corresponds to duplication of the same parameters through clones.

To go further and identify the key parameters that impact the change in tumor size and the resulting time to progression, we performed a sensitivity analysis on all individual patients characteristics (Fig. 7).

Both analyses on tumor radius and TTP consistently identified the immune system (2 parameters), neo-angiogenesis (1 parameter), tumor initial size (2 parameters), initial size of the resistant subclone (1 parameter), as well as 1 parameter encompassing the impact of implicit mutations on cell proliferation cancer hallmark as critically impactful on both outputs of interest.

## Discussion

The in silico *EGFR*-mutant lung adenocarcinoma (ISELA) model presented in this paper is a predictive and reliable mechanistic model of tumor growth evolution for patients treated with gefitinib with TTP as primary outcome. This model includes patients’ individual characteristics variability observed in literature. The model was designed with knowledge and data available in public literature. The calibration outcome and the corresponding visual predictive checks show the successful calibration with more than 85% of patients accurately reproduced. Visual predictive checks are a valuable tool widely used in the field of modeling in particular in pharmacodynamics^25,26^, which helped build credibility for the calibrations performed. To go even further, and to assess the credibility of the ISELA for prediction, a formal validation of the model output with respect to an independent dataset showed agreement with the real world clinical data. These results underline the capacity of the model in predicting tumor progression in a population of patients with *EGFR*-mutant LUAD treated with gefitinib.

The predictive accuracy of the model has been validated on population-based data extracted from ref. ^23^, based on two metrics: bootstrapped log-rank tests (more than 99% of tests were negative) and clinical data coverage (more than 98% of coverage was observed). As a consequence of this validation, the ISELA model predictions are deemed reliable on *EGFR* mutant LUAD patients at the population level, for patients that do not experienced severe toxicity, death, or treatment discontinuation.

One advantage of the mechanistic approach is that each parameter holds a pathophysiology-related meaning: causality between disease-related biological phenomena is inherent to the knowledge-based model, easing the interpretation of the impact of parameter values on clinical outcomes, especially interesting in the context of uncommon populations. Exploration of rare populations was therefore realized, and we compared *EGFR* mutant LUAD population with and without *KRAS* mutation: the obtained results were in line with reported knowledge, namely (i) consistency in the population characteristics: *KRAS* is an uncommon mutation, around 2.5% reported on trials based on a population with *EGFR* mutant LUAD^27^ and 2.35% in the virtual population defined earlier; (ii) consistency in the efficacy of gefitinib in *KRAS* and *EGFR* mutant LUAD: first-generation *EGFR*-TKIs, i.e., gefitinib and erlotinib transiently down-regulates also the activity of mutant KRAS and related downstream signaling pathways^24^; (iii) consistency in TTP of *KRAS* and *EGFR* mutant LUAD patients: Patients harboring *KRAS* mutations are associated with a shorter time to progression during TKI-treatment^24^. Being able to reproduce behaviors that were not the focus of this study increases the credibility of to the model.

The ISELA model can be further improved. Currently, individual behavior description should be interpreted with caution, as some characteristics were not available at the individual patient level, thus correlations between descriptors were extrapolated from the calibration process. To the best of our knowledge, it is difficult to access individual data on tumor size evolution over time, tumor mutational burden (e.g., number of driver mutations, number of clones in the tumor), and individual patient characteristics (e.g., age, sex). Access to such individual-based data would improve the calibration process and the predictions made at individual level. Sensitivity analysis of the model identified neo-angiogenesis and immune-related phenomena as the two main drivers of TTP progression and tumor size. These parts of the model currently remain phenomenological. However, they could be further detailed as part of the future development of the ISELA model, in order to better study mechanistically how these phenomena impact on clinical outcomes. This would also help to increase the domain of applicability of the model.

The model could be extended to new contexts of use taking into account new mutations or new treatments and thus be adapted to support several drug development lines. One advantage is that the ISELA model was planned and implemented to allow enhancements as scientific knowledge progresses. Both qualitative and quantitative advances can be used. As a consequence, if new relevant information is found regarding the physiopathology, it can be integrated in the existing model, rather than rebuilding a model from scratch. Finally, to further explore the advantages and drawbacks of the ISELA model one could compare it with mathematical models applied to the same context of use: patients with *EGFR*-mutant lung adenocarcinoma treated with first-generation TKI.

In silico approaches such as the one presented in this article provide tools to overcome frequent issues related to clinical trials: they notably ensure the clinical equipoise by enrolling the exact same virtual patients in control and investigational arms. As a consequence, in silico models supporting drug development can ease the development of new drugs improving the medical care of patients diseases such as LUAD^28,29^.

## Materials and methods

### Development of the ISELA model

**Fig. 8:**
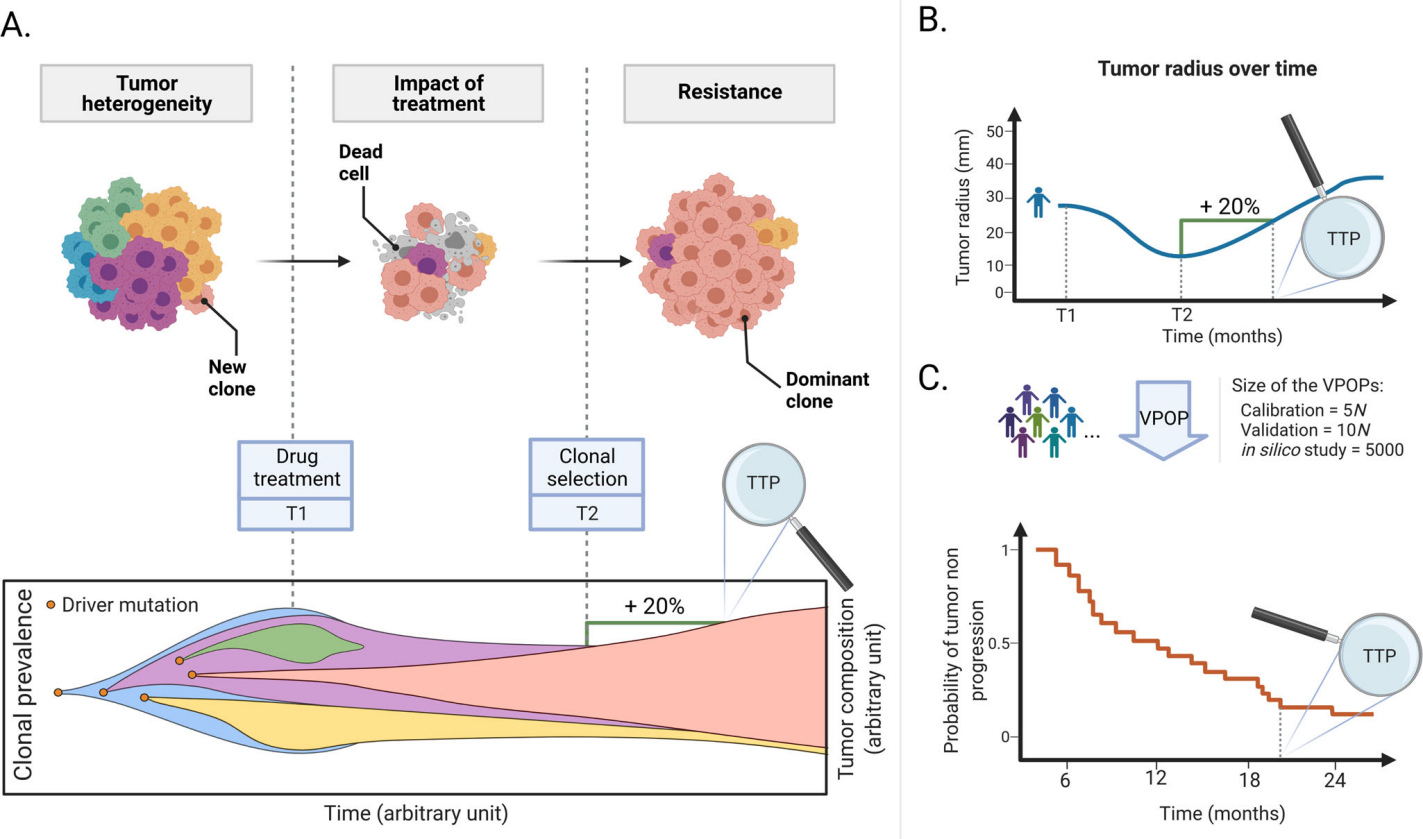
Quantification of tumor size evolution affected by clonal prevalence. **A** Tumor growth and heterogeneity. A solid tumor can be seen as a group of tumor clones that harbor different phenotypes due to specific clone mutations. Upon drug administration, some clones will shrink and may be destroyed, while others will resist and become dominant. By following the size of each clone, one can deduce the volume of the tumor and its radius, and therefore the time to progression (TTP) according to the RECIST criteria (i.e., increase of 20% of the size of the tumor radius). **B** Tumor radius evolution computed on each virtual patient (Box 1) and the time to progression is deduced from RECIST criteria. **C** Tumor radius is followed for each patient of the virtual population (Box 1) and represented with a Kaplan–Meier visualization of the probability of tumor non-progression (orange). N is the number of patients in the comparator experimental dataset. VPOP: virtual population; TTP: time to progression. Created with BioRender.com.

The ISELA model is a knowledge-based mechanistic model designed to reproduce tumor size evolution and disease progression of virtual patients matching real world patients with *EGFR*-mutant LUAD treated with gefitinib, as illustrated in Fig. 8. Together, virtual patients form virtual populations (see Box 1). The clinical outcome deemed of interest is the time to progression based on RECIST (Response Evaluation Criteria In Solid Tumors) criteria^30^. Briefly, this corresponds to an increase of the largest dimension of the tumor by 20% and of at least 0.5 cm.

A thorough review of more than 250 scientific papers was performed to identify the main phenomena to include into our EGFR mutant LUAD physiopathology model: (1) cell proliferation, cell death, layering of cells in the tumor, carrying capacity due to neo-angiogenesis and limited growth due to the immune system impacting tumor growth, (2) impact of individual mutational profile on these pathways, (3) signaling pathways that are downstream of *EGFR* activation, (4) tumor heterogeneity stemming from groups of cells sharing the same phenotype, namely tumor clones; and (5) resulting clinical outcomes from physiopathology (Supplementary Information). Due to the model modularity, (6) a gefitinib treatment model was added in order to consider its impact on patients’ physiopathology as described in Table 2. The associated knowledge was validated based on thoracic oncology scientific expertise. This allowed us to uncover the knowns and unknowns of the target population characteristics, and define appropriate simplifying assumptions when needed. This biological knowledge was converted into mathematical equations (ordinary differential equations—ODEs) to computationally model the corresponding biological phenomena. These equations were implemented as groups of mechanistically related equations, or submodels, and the integrated combination is the ISELA model.

**Table 2:** ISELA submodels specificities.

	Submodel name	Included biological phenomena	Submodel components
1	Tumor growth	Cell proliferation, cell death, layering of cells in the tumor, carrying capacity due to neo-angiogenesis and limited growth due to the immune system impacting tumor growth	Variables: 5 X *N*
			Parameters: 15
			ODEs: 5 X *N*
2	Tumor mutational profile	Mutational burden of the tumor	Variables: 0
			Parameters: 32+4 X *N*
			ODEs: 0
3	EGFR signaling pathway	Transduction of cell proliferation, cell survival signals	Variables: 4
			Parameters: 28+6 X *N*
			ODEs: 0
4	Tumor heterogeneity	Heterogeneity (co-existence of several distinct clones in the tumor)	Variables: 0^a^
			Parameters: 0^a^
			ODEs: 0^a^
5	Clinical	Disease outcomes	Variables: 9
			Parameters: 7
			ODEs: 3
6	Gefitinib treatment	Pharmacokinetics (PK) and mechanism of action of gefitinib	Variables: 4
			Parameters: 17
			ODEs: 0

The number of clones *N* represented can vary from 2 to 16.

*ISELA* In Silico EGFR mutant LUAD, *ODEs* ordinary differential equations, *EGFR* epidermal growth factor receptor.

^a^Phenomena are taken into account by duplication of other submodels (variables, parameters, and ODEs).

**Fig. 9:**
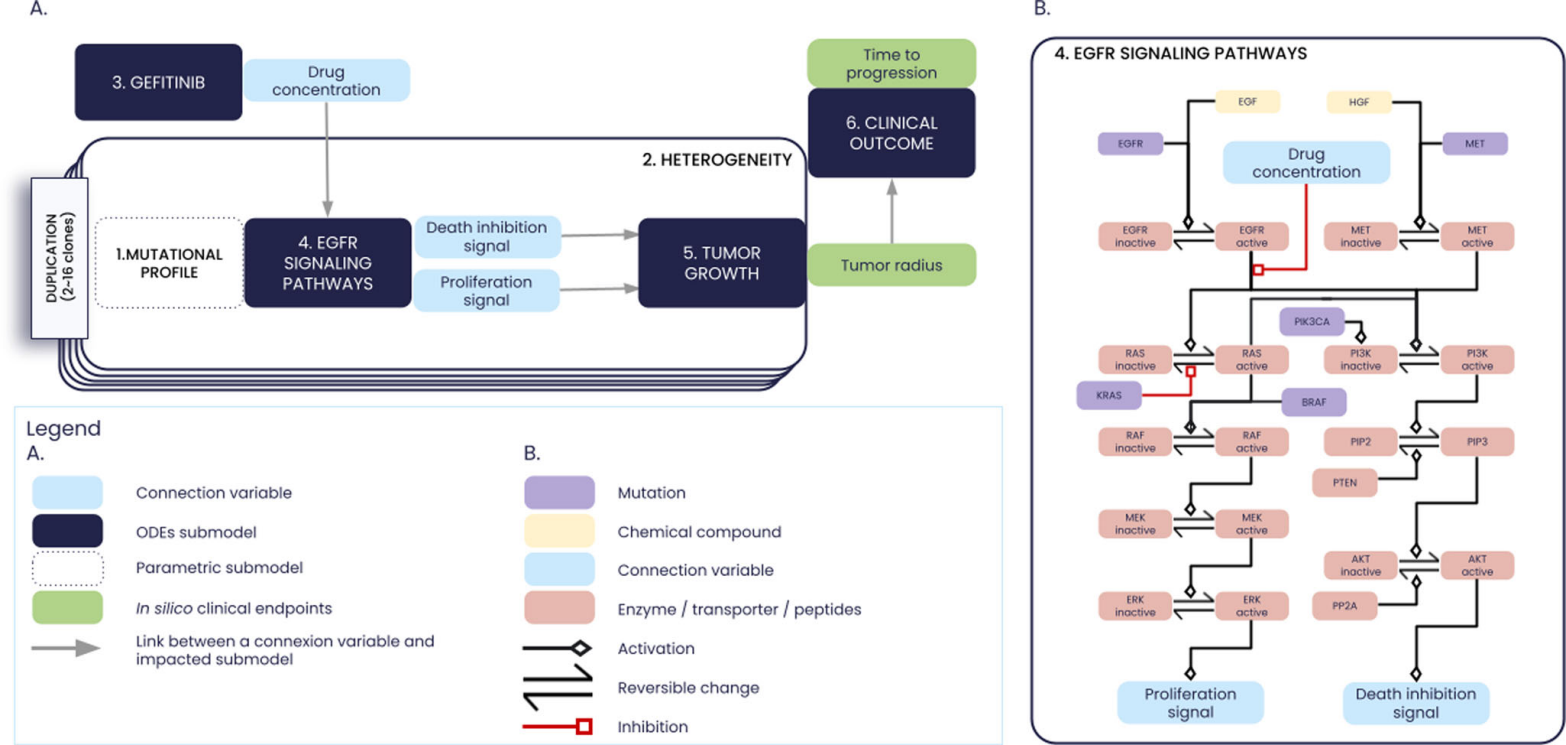
Illustration of the In Silico *EGFR* mutant LUAD (ISELA) model. **A** Structure of the ISELA model: the different submodels of the ISELA model (see Table 2) are labeled and their connecting variables are represented in light blue. The two main model outputs are also represented (i) the biological one, corresponding to the radius of the primary tumor; (ii) the clinical one, corresponding to the time at which the tumor progressed in size, according to the RECIST (Response Evaluation Criteria In Solid Tumors) criteria. **B** Focus on the *EGFR* signaling pathways submodel: graphical representation of the *EGFR* signaling pathways variables corresponding to chemical compounds (yellow) to intracellular proteins (light red) that link the connection variables (light blue). Mutations that impact the pathways are also represented in purple.

The ISELA model accounts for the heterogeneity in tumor with the modeling of a number of tumor clones with distinct genetic background from one patient to another. From a computational point of view, this is ensured by the duplication of each part of the model corresponding to phenomena occuring at the clone level. Namely, the following phenomena are duplicated to account for clone heterogeneity: tumor growth, mutational profile, EGFR, and downstream signaling pathways. As a consequence, depending on the number of clones each virtual patient carries in their tumor, the model runs with a set of 27 to 97 variables, 108 to 258 parameters, and 13 to 83 ODEs (ranges for 2 to 16 clones and associated duplication), as indicated in Table 2. The model structure is illustrated in Fig. 9, and the equations of the model are provided in Supplementary Information.

As indicated in Fig. 9, two model outputs are considered as clinical endpoints: tumor radius and time to progression, deduced from the tumor radius. Yet, the model does not consider censoring due to toxicity, death, or treatment discontinuation. Patients who did not display tumor progression at the follow-up cut-off, that is to say at the end of the simulation, may be considered as being right censored.

### Model calibration

Following the model development detailed in the previous section, the model was calibrated as advised in the EMA guidelines^20^ and the V&V 40^21^. Calibration aims to find parameter values and distributions such that the model reproduces expected behaviors observed in the real world. It is the first step to ensure the accuracy of a mechanistic model and is performed prior to the validation process. We here describe the calibration protocol applied to the ISELA model, based on the data we found in literature. The corresponding calibration process is composed of successive steps, and each step has as its objective a specific model variable behavior matching one or more specific computational constraints. Since calibration steps are executed sequentially, the first calibration steps are prerequisites for the following steps. They take into account both quantitative and qualitative constraints, to consider and reproduce the heterogeneous and multi-scale data extracted from literature^31^ details the two first steps of the process, where we aligned the ISELA model with:

- published in vitro dynamics to calibrate EGFR/cellular mesenchymal epithelial transition (cMET) associated pathways and tumor growth in vitro^32–35^.
- published xenografted mice data (see Table 3)^36–39^ to calibrate tumor growth in xenograft mice.

In addition, we performed two additional calibration steps to increase the context of use of the ISELA model to humans They both focus on finding values of the parameters related to human neo-angiogenesis, immune system, and treatment-resistant clones to reproduce the time to progression (TTP; i.e., duration between start of the treatment administration and detection of tumor progression) of patients found on literature:

- We reproduced individual clinical data (time to progression) found in literature^40–45^, where patient characteristics such as gender and type of *EGFR* mutant mutations are provided.
- We reproduced the population-level clinical data deduced from the NEJ002 trial^46^, and deduced correlations between patient descriptors. The extraction of the list of time-to-events (for both PFS and OS) was realized using R package digitize^47^, using the input survival times from graph reading; and the reported number at risk. TTP was inferred based on the clinical trial PFS and OS, as detailed in ref. ^22^. Therefore, the NEJ002 TTP dataset was deduced from the lists of time-to-events corresponding to the PFS and OS of the Maemondo/NEJ002 trial. Under the hypothesis that patients who died before disease progression are characterized by the same time to event in the PFS and OS sets, we filtered out PFS events that correspond to patients’ death, leaving only the time-to-events corresponding to disease progression.

These two last steps were not intended to reproduce tumor size evolution over time (as done in steps 1 and 2) since in vivo tumor sizes are rarely reported in the literature in humans. Instead, the goal was to reproduce the TTP, computed from the evolution of the time tumor progression, according to the RECIST criteria. The experimental data that were used in these four calibration steps are listed in Table 3.

**Table 3:** References used for the calibration process, focusing on implemented constraints on tumor size.

Author (year)	Study type	Biological processes
^32^	In vitro	Intracellular pathway phosphorylation profiles
^33^	In vitro	Intracellular pathway phosphorylation profiles
^34^	In vitro	Intracellular pathway phosphorylation profiles
^35^	In vitro	Intracellular pathway phosphorylation profiles
^36^	In vitro	Ex vivo
^37^	Ex vivo	Evolution of the proportion of proliferative cells within the tumor
^38^	In vivo	Estimation of the viable rim size^a^ of the tumor
^39^	In vivo	Estimation of tumor volume
^40^	Clinical data	Time to progression
^41^	Clinical data	Time to progression
^42^	Clinical data	Time to progression
^43^	Clinical data	Time to progression
^44^	Clinical data	Time to progression
^45^	Clinical data	Time to progression
^46^	Clinical data	Time to progression

^a^Rim size = distance between tumor core and tumor surface.

To use the same model structure in all settings (in vitro, in mouse, and in human simulations), allometric scaling was used, as described in ref. ^31^. In a nutshell, allometry theory refers to the impact of the size of living creatures on their characteristics such as morphological and physiological traits. In this paper, we used a common scaling law with the following relationship *Z* = *a* × *M*^*b*^ with *Z* the studied characteristic, *M* the organism mass, and *a* and *b* parameters called allometric coefficient and allometric exponent, respectively^48,49^. As reference weight for in vitro and mice, we used 2.63 g^50^ and 23 g^51^, respectively.

Visual predictive checks are performed as a verification criterion of calibration success (see “Results” section).

### Model validation

As detailed in the next two sections, model validation was assessed on the Lux-Lung 7 clinical dataset, and based on the simulation of a virtual population matching its characteristics.

#### Validation dataset

The Lux-Lung 7 trial (with PFS and OS reported by ref. ^23^) was selected for three reasons:

- The characteristics of the patients enrolled in the trial corresponds to the specified context of use of the ISELA model,
- The treatment they received (gefitinib) is consistent with the context of use of the model,
- The dataset was neither used for building nor calibrating the ISELA model.

These are reported in Table 4.

**Table 4:** Characteristics of the LUX-LUNG 7 population, reported by Paz-Ares et al.^23^.

Characteristics	Lux-Lung 7
*Gender, n (%)*	
Men	53 (33.3)
Women	106 (66.7)
Age in years - median (range)	63 (36–89)
*EGFR mutation*	
Exon 19 deletion (%)	93 (58.5)
Exon 21 L858R point mutation (%)	66 (41.5)^a^
*Smoking status*	
Never smoker (%)	106 (66.7)
Former smoker (%)	19 (11.9)^b^
Current smoker (%)	34 (21.4)^b^
*Ethnicity*	
Asian (%)	88 (55)
Non-asian (%)	71 (45)
*Clinical stage at screening*	
IIIb (%)	3 (1.9)
IV (%)	156 (98.1)

Details from smoking status were retrieved from ref. ^52^ also reporting analyses from the Lux-Lung 7 trial.

^a^One of the patients with L858R was reported as having an additional mutation in ref. ^52^ but not in ref. ^23^. For Vpop comparison this virtual patient was considered in the L858R category.

^b^Former smokers are considered as light ex former smokers from the paper; while current smokers are considered as other current or former smokers from the paper.

**Fig. 10:**
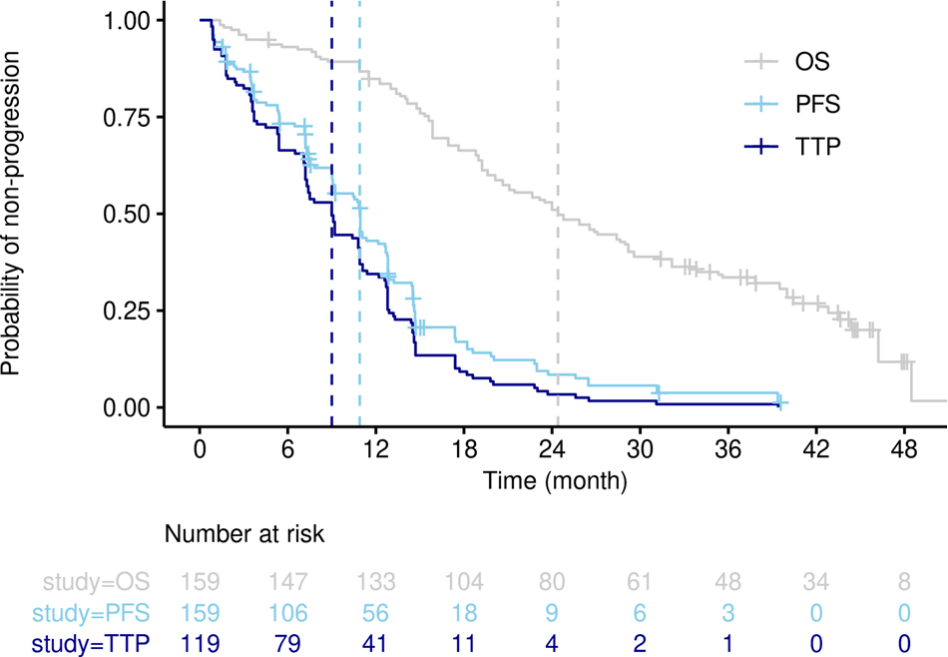
Overall survival (OS, gray), progression-free survival (PFS, light blue), and time to progression (TTP, dark blue curve) from the Lux-Lung 7 dataset. TTP corresponds to the PFS curve after removal of dead and censored patients. Median OS (24.4 months), PFS (10.9 months), and TTP (9.0 months) are represented with dotted lines highlighting the effect of data-process on time corresponding to the median probability. OS and PFS data were manually extracted from ref. ^23^, processed and plotted in R, version 3.6.3, with the packages survival (version 3.1-8) and survminer (version 0.4.8).

To be able to compare the ISELA model TTP to the LUX-LUNG 7 dataset, the disease progression endpoint was similarly derived from clinical PFS and OS, as explained in ref. ^22^ and detailed in the calibration context. Therefore, the Lux-Lung 7 TTP dataset was deduced from the lists of times-to-event corresponding to the PFS and OS of the Lux-Lung 7 trial. The comparison of OS, PFS, and TTP is provided on Fig. 10. In the absence of information about which patients did not display tumor progression (and died without detectable progression), we assume that the distribution of patients characteristics is not altered in the subsets of patients who displayed tumor progression.

#### Virtual population generation and statistical analyses for validation

The protocol described in Table 5 was applied to compare the simulated output with the clinical data set.

**Table 5:** Summary of the 5-step validation protocol.

Step name	Process
1. Generation of the virtual population	Known distributions of patient baseline characteristics are extracted from available data, indicated on Table 4.
	When information was only partial, some assumptions were made in order to obtain a well-defined distribution:
	• Standard deviation was estimated from quantiles when not available.
	• We assume that the population only contains common EGFR mutations (exon 19 deletions and L858R point mutation).
	For patients and population characteristics not reported in ref. ^23^ (e.g., preponderance of KRAS (standing for K isoform of rat associated sarcoma protein) mutation, proportion of patients with mutation co-occurring with EGFR mutations, exact TNM (tumor, lymph node, and metastatic) status), distributions and correlations identified on the general population (from refs. ^27,53–56^) and from calibration steps 3 and 4 were used.
	The generated virtual population was 10 times bigger than the real population, to allow bootstrapping and estimation of a prediction interval (PI). For all virtual patients, duration of follow-up, which corresponds to the maximal simulated time, corresponds to the period reported in the experimental clinical dataset.
2. Comparison of Virtual and real population	For each patient characteristic, we compared baselines between Virtual population and real population from LUX-LUNG 7 dataset: statistical comparisons are performed using the Fisher test for discrete characteristics and the t-test for continuous ones to ensure that baselines are indeed not statistically different.
3. Kaplan–Meier curves visualization	The TTPs of simulated patients are plotted in Kaplan–Meier visualization in R (built-in survfit function).
4. Computation of prediction interval	The bootstrapped 95% PI of the simulated Kaplan–Meier curve was computed based on 1000 subsamples from the Virtual Population for convergence, each sub-sampling having the same sample size as the real population.
5. Computation of validation metrics	The final validation metrics are the following:
	Raw coverage:
	The raw coverage is defined as the percentage of the Kaplan–Meier curves extracted from ref. ^23^ that lie within the prediction interval around the simulated curve in the Kaplan–Meier visualization. A validation threshold was set to a coverage of 80% of the observed curve by the PI.
	Bootstrapped log-rank test:
	The bootstrapped log-rank (LR) test consists of performing multiple LR tests, each comparing TTP distribution between the one from a sampled population taken from the Virtual population, namely bootstrapped population, with the TTP distribution of the experimental dataset. The virtual bootstrapped population size used for each test was equal to the size of the related real population. We computed 5000 LR tests as advised in ref. ^22^ and added 2000 tests to ensure convergence. If the percentage of statistically non-significant bootstrapped tests (*p*-value > 0.05) is greater than a given threshold, the model is considered able to reproduce the observed results. A validation threshold was set to 80% negative LR tests.
	The model is considered as validated if both validation metric scores are above pre-defined thresholds.

The data processing and analysis were performed within R Software, version 3.6.1 or above. In particular, we used the following packages: survival, survminer, tidyr, data.table, jsonlite, and ggplot2.

#### Sensitivity analysis

We chose to perform sensitivity analysis based on a tornado approach. In a nutshell, a population of 5000 virtual patients was generated, based on the characteristics of the general population (Supplementary Information), a 50/50 proportion of EGFR mutations (exon 19 deletions and exon 21 L858R point mutation) was used. For each patient characteristic, patients were split in two categories: low value (those with value lower than the median) and high value (those with value higher than the median) and the median output of interest was computed for each category. The value used for the comparison of all parameters is the difference between the median output of interest in the complete virtual population minus the median output of interest in each of these two categories. The resulting values are plotted in tornado plots. The advantage of such an analysis is that it does not rely on statistical hypotheses on the distribution of the impact of the parameter on the output of interest.

### Reporting summary

Further information on research design is available in the Nature Research Reporting Summary linked to this article.

## Supplementary information

Supplementary Information

Reporting Summary

Supplementary material - source files

## Data Availability

The model structure, model documentation, and data supporting the conclusions of this study are available on the jinko.ai platform upon request from the corresponding author, C.M.

## Code availability

Model documentation and source code files for submodels are provided in Supplementary Information.
