# Supplementary material for: Knowledge-based mechanistic modeling accurately predicts disease progression with gefitinib in EGFR-mutant lung adenocarcinoma
Source: NPJ Syst Biol Appl. 2023 Jul 31;9:37. doi: 10.1038/s41540-023-00292-7 (PMC10390488; doi:10.1038/s41540-023-00292-7)
Supplement: Supplementary file 1 — Supplementary Information [file 41540_2023_292_MOESM1_ESM.pdf]

# Supplementary Information
## Knowledge-based mechanistic modeling accurately predicts disease progression with gefitinib in *EGFR*-mutant lung adenocarcinoma

Adèle L'Hostis[1†], Jean-Louis Palgen[1†], Angélique Perrillat-Mercerot[1], Emmanuel Peyronnet[1], Evgueni Jacob[1], Jim Bosley[1], Michaël Duruisseaux[2,3,4], Raphaël Toueg[5], Lucile Lefèvre[5], Riad Kahoul[1], Nicoletta Ceres[1] and Claudio Monteiro[1*]

[1*]Novadiscovery SA, Pl. Giovanni da Verrazzano, Lyon, 69009, Rhône, France.
[2]Respiratory Department and Early Phase, Louis Pradel Hospital, Hospices Civils de Lyon Cancer Institute, Lyon, 69100, France.
[3]Cancer Research Center of Lyon, UMR INSERM 1052 CNRS 5286, Lyon, France.
[4]Université Claude Bernard Lyon 1, Université de Lyon, France.
[5]Janssen-Cilag, France, 1, rue Camille Desmoulins - TSA 60009, Issy-Les-Moulineaux Cedex 9, 92787, FRANCE.

*Corresponding author(s). E-mail(s): claudio.monteiro@novadiscovery.com;
†These authors contributed equally to this work.

# Supplementary material A – ISELA model documentation

The ISELA model is a knowledge-based mechanistic model designed to reproduce tumor size evolution and disease progression of virtual patients matching real-world patients with EGFR-mutant LUAD treated with gefitinib. The ISELA model is made of six submodels: (i) Tumor Growth, (ii) Tumor mutational profile, (iii) EGFR signaling pathway, (iv) Tumor heterogeneity, (v) Clinical submodel, (vi) Gefitinib treatment. For all these submodels, a detailed documentation was extracted from jinkō.ai platform and presented below.

The implementation of these models is also provided in SBML (L3V2), XLSX and ANT format. These files were generated using the `libAntimony` tool (v2.13.2) (Medley et al, 2018; Choi et al, 2018).

## A.1 Tumor Growth

A solid tumor can be seen as a group of tumor clones, where a tumor clone is a group of cells that harbor the same phenotype due to specific clonal mutations (see section A.2.1 for more details). Growth of the tumor clones is modeled with the Tumor Growth submodel, that focuses mainly on the growth and proliferation of tumor cells by describing three cell subpopulations inside the clone: proliferating cells, quiescent cells and dead cells.

A few hypotheses lie behind this modeling choice. First, we hypothesize that the supply of resources needed by the cells such as nutrients is perfectly correlated with oxygen supply: this assumption allow us to consider oxygen as a proxy of nutrients availability. Then, we also assume that the oxygen profile from the outside to the inside of the tumor is continuously decreasing, from physiological values at tumor edge. Then, by fixing two threshold values for oxygen concentration (such that both proliferating state and alive state are determined by two distinct oxygen concentration thresholds), we are able to consider two spatial depths thresholds in order to create three subpopulations implicitly taking nutrients into account.

Each tumor clone will therefore have its own set of proliferating and quiescent cells; but as the distinction is not relevant, a unique pool of dead cells is modeled, regardless of their original clone, as illustrated in the Supplementary Figure A.1, representing the cellular dynamics of a given clone.

### A.1.1 Tumor geometry and model structure

We assume that the tumor is a perfect sphere with a central symmetry. The state of a tumor radius is thus representative of the entire tumor. The tumor volume can then be easily derived from the tumor radius. We postulate that an unknown oxygen concentration profile (or gradient) exists across that tumor radius, which may dynamically change with tumor size evolution. This

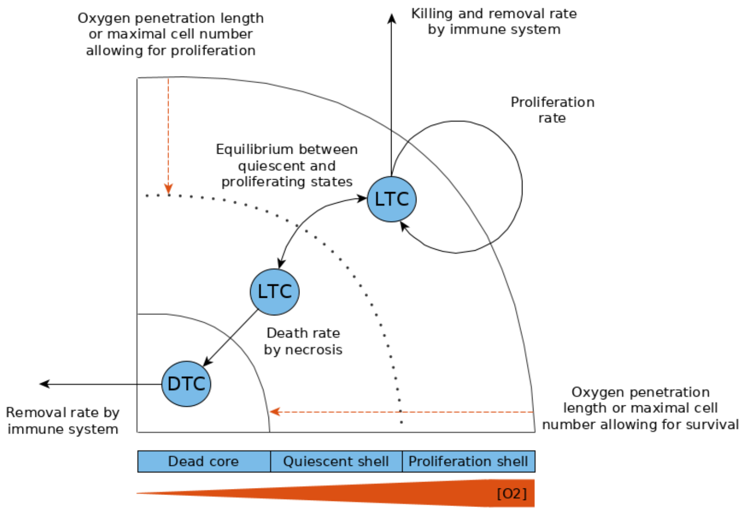


**Supplementary Figure A1** Graphical representation of the computational model of tumor growth. LTC: Living tumor cells. DTC: Dead tumor cells.

profile is governed by diffusion and vasculature (transport) properties. One assume that this profile is continuous and monotonically decreasing from the outside of the tumor where oxygen concentration is at physiological levels, to the center.

We also assume that all vital resource supply such as nutrients for the cells are perfectly correlated with oxygen supply, *i.e.* nutrients are always available to same extent as oxygen concentration. Under this hypothesis, one can use the oxygen only as a proxy for all resources and consider it as the single limiting resource.

We then defined a cell type $C_i$ as the set of cells that benefits from an oxygen concentration in their direct environment between values $o_{i-1}$ and $o_{i+1}$. By construction, if $o_0$ is the minimal oxygen concentration in the tumor and $o_{N+1}$ the maximal oxygen concentration in the tumor, then all tumor cells belong to one of the $N$ cell types defined in that way.

Based on this definition and our previous hypothesis on the oxygen profile, cell types are naturally ordered on the axis defined by the tumor radius and form distinct populations along it. Given this unidimensional geometry, each cell from a given type $C_i$ population can only interact with its neighbors (except for the border cell type populations $C_0$ and $C_N$) $C_{i-1}$ and $C_{i+1}$.

We also hypothesize that the cell density within each cell type $C_i$ is equal.

We also assume that the non-tumor cells are negligible and are therefore not taken into account for the computation of the size of the tumor.

We then make the hypothesis of the existence of the two following oxygen concentration thresholds:

- Threshold $T_1$ under which tumor cells do not have enough oxygen to live so that die by necrosis.
- Threshold $T_2$ under which tumor cells do not have enough oxygen to proliferate so that they pause the cell cycle.

This hypothesis implies that these three proliferating, quiescent (living but not proliferating) and dead states depends on the oxygen concentration and that unique and well-defined thresholds exist to clearly segregate any cells between these three states. This also implies that the transition between these states is instantaneous without any intermediate or transient state.

As these thresholds are shared by all cells, independently of their state or origin, they can be considered as an extrinsic biological property.

As proliferating cells are alive, we deduced that $T_1 \leq T2$ since proliferating cells are alive.

Based on these two thresholds, we defined $N = 3$ cell types, using $o_1 = T_1$ and $o_2 = T_2$, that have the following characteristics:

- $C_1$ cells are dead and cannot proliferate. They are called "dead" cells.
- $C_2$ cells are alive but can not proliferate. They are called "quiescent" cells.
- $C_3$ cells are alive and can proliferate. They are called "proliferating" cells.

As the oxygen concentration profile is assumed to be monotonically decreasing from the spherical tumor surface to its center, there can be determined equivalent depth thresholds characterizing at which depth within the tumor these oxygen concentration thresholds are reached. Then, assuming the tumor density is constant, this is equivalent to define critical cell numbers above which the tumor radius will be greater than these depth thresholds. We thus define:

- The length $l_1$ such that all cells located at a lower depth from the tumor surface receive oxygen in levels greater than $T_1$ threshold level, and are thus proliferating ; or equivalently, the maximal number of cells $N_1$ that can be located within $l_1$ from the tumor surface
- The length $l_2$ such that all cells located at a lower depth from the tumor surface receive oxygen in levels greater than $T_2$ threshold level, and are thus alive ; or equivalently, the maximal number of cells $N_2$ that can be located within $l_2$ from the tumor surface.

It should be noted that $N_1$ and $N_2$ necessarily depend on the tumor radius, assuming constant cell density, since the larger the tumor the more

cells can fit in a $l_1$ or $l_2$ layer under the surface.

We chose not to model explicitly the spatial oxygen profile within the tumor by parameterizing the submodel in terms of spatial depth (or the equivalent view in terms of critical cell numbers) that implicitly takes into account the oxygen profile properties. These threshold parameters also implicitly take into account the mechanisms linked with tumor vasculature. Some specific processes such as neoangiogenesis are explicitly taken into account by modulating these parameters (see section A.1.4).

The submodel structure thus consists in a spherical tumor composed of a dead cell center, surrounded by a middle layer of quiescent cells, themselves surrounded by an external layer of proliferating cells. The model main variables are the number of cells within these three population which are dynamically described by considering the interactions between the cell types, the transition of cells from one type to another one, and the interactions of proliferating cells with their external environment. These different interactions or processes that are considered in the submodel are described in the following subsections.

### A.1.2 Cell proliferation

Tumor Growth results from cell proliferation. The tumor is divided into two types of cells: dead cells modeled with the variable `DeadTumoralCells` and living cells with the variable `LivingTumoralCells`. As described with submodel structure, living cells may proliferate only if they are close enough to the tumor such that they receive enough oxygen. This maximal number of cells $N_1$ that can proliferate depends on tumor vasculature and is denoted with the parameter `maxProlifCellsWithAngio` when neoangiogenesis mechanisms are involved and with the parameter `maxProlifCellsWithDiffusion` otherwise. When the number of living cells is lower than this critical threshold, all living cells may proliferate, otherwise, only as many as cells as this critical number undergo proliferation.

We assumed that the transition between these quiescent and proliferating state is much faster in front of the other time scales in our model, that is can be modeled as instantaneous. The short time scales needed to describe cell cycle are not relevant to describe overall tumor size evolution.

Proliferation is modeled with saturated first-order kinetics, such that a single parameter characterizes the proliferation rate. The saturation was introduced to prevent the entire tumor from growing above a critical size called carrying capacity. This carrying capacity is set at the lung volume such that tumor size can not reach biologically unrealistic levels.

The proliferation rate is also modulated with a Hill function that depends on the EGFR signaling pathway output proliferation signal, parameter

`cellProlifEqProliferating` in our model (see section A.4), in order to bridge intracellular signaling to cell behavior.

### A.1.3 Cell death by necrosis

Quiescent cells that do not receive enough oxygen die by necrosis and their debris contribute to the formation of the necrotic dead core at the center of the tumor.

In a similar fashion proliferating cells were defined, cells are considered to receive enough oxygen to live as long as they remain above a critical cell number $N_2$. This thresholds parameter depends on tumor vasculature and is denoted with the parameter `maxLivingCellsWithAngio` when neoangiogenesis mechanisms are involved and with the parameter `maxLivingCellsWithDiffusion` otherwise.

When the number of cells is greater than this critical threshold, cells start to die by necrosis. In this case, the death rate is assumed to be proportional to the difference between the total cell number and this critical threshold. When cells die, the tumor necrotic core increases by as much volume than previously occupied by these cells.

### A.1.4 Neoangiogenesis

Oxygen delivery is reduced in the inner regions of the tumor, and the distance on which oxygen has to diffuse increases with tumor growth, reducing oxygen availability for tumor inner cells. Neoangiogenesis is a mechanism promoted by the tumor to overcome this oxygen shortage by inducing vasculature development in the tumor.

As previously defined, oxygen diffusion is accounted for in our model by two critical thresholds ruling if cells receive enough oxygen to either live and proliferate. In the absence of neoangiogenesis processes, we assumed that the maximal depth at which cells receive enough oxygen to survive is about 104 µm *in vitro*. With the constant cell density assumption, this allowed to derived a value for parameter `maxLivingCellsWithDiffusion`. We then made the assumption that the maximal number of cells that can proliferate, the parameter `maxProliferatingCellsWithDiffusion`, is a constant fraction of this former threshold.

We modeled neoangiogenesis as an additional mechanism that increases these two critical cell numbers, as neoangiogenesis allows oxygen to be available to more cells or in higher levels. The parameter accounting for the maximal number of living cells in the tumor `maxLivingCellsWithNeoangiogenesis` is thus assumed to be equal to `maxLivingCellsWithDiffusion` plus a parameter `extraLivingCellsWithAngio` that is much higher than the former one as neoangiogenesis-induced vasculature is assumed to be a

more efficient mechanism than diffusion for delivering oxygen inside the tumor. `extraLivingCellsWithAngio` is constant in one patient, but can vary from one patient to another. Similarly as the modeling without neoangiogenesis, we assumed that the maximal number of cells that can proliferate taking int account neoangiogenesis, the parameter `maxProliferatingCellsWithNeoangiogenesis`, is a constant fraction of `maxLivingCellsWithNeoangiogenesis`.

### A.1.5 Immune system

The ISELA model focuses on late cancer stages, hence when the immune system - tumor setting is likely to be close to an equilibrium. We thus decided set the number of immune system cells constant in the tumor micro-environment.

## A.2 Tumor heterogeneity

This submodel aims at accounting for the tumor heterogeneity that is characterized by the diversity of mutations, genotypic alterations and phenotypic modifications that the restricted set of driver mutations explicitly modeled can not represent (see section A.3). The tumor heterogeneity submodel thus adds more complexity to the Tumor Growth submodel in order to account for this heterogeneity observed in real tumors.

The rationale to add more complexity to the Tumor Growth submodel is that tumors may be composed of different regions that growth and respond to treatment in different extent. In particular, if all cells in the tumor were homogeneous in our model, if a treatment-induced resistance alteration appears (see section A.2.2), all cells would benefit from it, and the tumor would instantaneously relapse, which was deemed tautological and unrealistic. We thus estimated that accounting for intratumor heterogeneity was necessary in order to represent more realistically the tumor composition and behavior. Then, if a resistance alteration would appear in a fraction of cells only, these cells would earn a proliferative advantage that would lead them to expand competitively, which is closer to the expected biological behavior.

### A.2.1 Tumor clonality

We thus decided to model a population of clones in the tumor, each clone being characterized by a different mutational signature compared to the other ones. In our model, the tumor cells are split in as many populations as the number of clones coexisting in the tumor.

Each of these populations is ruled by the equations of the Tumor Growth submodel and EGFR pathway submodel, which are thus duplicated for each clone. Each clone is assumed to be an independent sub-tumor, driven by these equations, not interacting directly with the other clones.

To account for clone interactions and integration of clones within an overall tumor, the following changes are made:

- The dead cell population was not duplicated for all clones, as these consists in necrotic debris that stand passively in the model, and their mutational profile is thus not impactful. The `DeadTumoralCells` variable is thus not duplicated and all quiescent cell dying are integrated to this common compartment independently of their properties.
- We assumed that the heterogeneous tumor keeps its perfectly separated dead and living layers, and that each cell type in each clone occupies an angular sector or fraction of the entire tumor type layer.
- When the tumor is made of more than one clone, transition rates between one cell type to the other are scaled not with the cell type surface, but with the portion of the cell type surface visible by the clone cell type. This thus add a new ratio to the rates equal to the number of cells of the clone of this cell type over the total number of cells of this cell type in this tumor. This correction is necessary to preserve the mass balance in this new model structure.

In addition to the number of clones for which we know the distribution from literature – thus a maximal number of 15 is created in the model – another clone is created, named the clone 0, in order to represent the clone that may acquire a resistance mutation and expand upon treatment administration.

To represent the heterogeneity between clones in our model, we identified parameters to account for the impact of unmodeled mutations on cancer hallmark mechanisms. The only exception is for the neoangiogenesis hallmark for which there no parameter defined differently between clones. These parameters may have different values between clones within the same tumor, and are listed below:

- `deltaCellProlifEq` parameter, which accounts for the impact of mutations on "proliferation" hallmark, as this parameter corresponds to the threshold of the Hill function used to link EGFR proliferation signaling to tumor proliferation rate.
- `deltaCellDeathInhibEq` parameter, which accounts for the impact of mutations on "cell death inhibition" hallmark, as this parameter corresponds to the threshold of the Hill function used to link EGFR death inhibition signaling to tumor death rate.
- `maxCellDeathInhibEq` parameter, which accounts for the impact of mutations on the "immune escape hallmark" as this parameter represents the maximal rate at which tumor cells may be killed by the immune system.

At the beginning of the simulation, each clone is assumed to share the same volume percentage of the initial tumor, i.e. they all have the same size.

### A.2.2 Treatment-induced resistance-conferring alteration apparition

After treatment initiation, a resistance-conferring alteration may expand in the tumor. These alterations are named treatment-induced alterations, as opposed to driver alterations, in order to easily distinguish them, although both can confer a proliferative or survival advantage to the tumor. The naming difference refers to the time of apparition of these alterations: driver alterations exist prior treatment application, whereas treatment-induced alterations appear afterwards.

We selected the following treatment-induced alterations to account for in our model:

- EGFR T790M mutation
- MET amplification
- HER2 amplification
- BRAF mutation
- PIK3CA mutation

It is a knowledge gap whether treatment-induced alterations appear due to selection of cells that initially possess the alteration and thus gained a competitive advantage, or whether it appears by acquisition of that alteration due to selection pressure. We tested several hypothesis and favored the latter "selection" hypothesis. At the beginning of the simulation, a subclone is created from a parent clone to share the same characteristics except that this subclone already possesses the resistance mutation. This subclone remains neutral but might be selected upon treatment administration. We also assume that this clone is randomly chosen between all the pre-existing clones with equal probabilities. The initial size of this subclone (i.e. the number of cells possessing this mutation) is called `resistantSubCloneInitialSize`. This resistant subclone is defined as the clone 0.

We thus added the following descriptors the Vpop, in addition to the descriptors listed in the Tumor heterogeneity subsection:

- The type of alteration induced by the treatment (see list above). This is described by the `treatmentInducedAlteration` parameter. However, we considered that patients can not have a treatment-induced alteration they already have as driver alteration.
- The number of cells initially possessing the resistance mutation. this is described by the `resistantSubCloneInitialSize` parameter, which was calibrated during the model building.
- The number of the parent clone from which the resistance subclone originates. This is described by the `resistantSubcloneParentNumber` parameter.

We made the hypothesis that EGFR T790M mutation can not appear in a patient already bearing an exon 20 insertion since both mutations are located on exon 20 and we found no co-occurence evidence in the literature. We also assume that, when two mutation resistance appears, they appear on the same clone.

## A.3 Tumor mutational profile

This submodel aims at describing the variability shown in the real population to account for in the virtual population used for simulations on the integrated model. The description of each virtual patient lung tumor notably includes genetic alterations, clone number and clone sizes. These characteristics should be representative, in terms of distributions and correlations with patient descriptors, of what is observed in the real population.

We decided to include in our model only the driver alterations for which the protein was explicitely modeled in our EGFR signaling pathway submodel and for which alteration incidence was available. Thus, the following alterations are modeled:

- BRAF
- EGFR
- KRAS
- MET
- PIK3CA

We made the hypothesis to consider that all of these alterations are actually mutations and not amplifications or other genetic abnormalities. Furthermore, we made the hypothesis that all of these mutations are clonal (i.e. present in all clones).

The list of the main patient descriptors included in the virtual population is the following:

- Driver gene mutations (see list above), which incidences and associated correlations were estimated from literature. This is described by the `isXmutated` parameters for all mutations X.
- Type of EGFR specific mutation (see list above) which incidence was estimated from the literature and associated correlations from CLCGP data. This is described by the `mutEGFR` parameter.
- Number of clones, which distribution was estimated from Hanjani's study, by assuming that clones represent mutational clusters. A discrete lognormal distribution was manually fitted to account for a mean number of clones of 5 and values ranging between 2 and 15. This is described by the `nClones` parameter.
- Age, which distribution and associated correlation were estimated from CLCGP data. This is described by the `age` parameter.

- Ethnicity, which distribution and associated correlations were estimated from SEER resumed data. This is described by the `ethnicity` parameter.
- Sex, which distribution and associated correlations were estimated from CLCGP data. This is described by the `isMale` parameter.
- Tumor extension parameter, which distribution was estimated from SEER resumed data. This is described by the `extTumor` parameter.
- Smoking status, which incidence was estimated from CLCGP data. This is described by the `smokingStatus` parameter.
- Tumor initial size, which distribution was estimated from CLCGP data. This is described by the `initialTNM` parameter.
- N category, which distribution was estimated from CLCGP data (see section A.5 for more details). This is described by the `initialTNM` parameter.
- M category, which distribution was estimated from CLCGP data (see section A.5 for more details). This is described by the `initialTNM` parameter.

### A.3.1 Impact of driver alterations

Driver alterations confer a selective advantage to the tumor cells and clones by impacting one or several tumorigenesis-related mechanisms.

The effect of each selected mutation is modeled as explained in the following:

- EGFR: 3 mutations are taken into account for EGFR: exon 19 deletion, exon 20 insertion, and exon 21 L858R point mutations. These mutations are implemented as having three different impacts:
  - EGFR mutations lead to constitutive activation of EGFR, therefore the downstream pathways of EGFR are activated as well. This is verified for exon 19 and 21 mutations and it is hypothesized in the literature that exon 20 mutations lead to the same effects.
  - EGFR mutations modify the affinity of gefitinib towards ATP (see `treatmentInducedAlteration` in section A.6).
  - EGFR mutations modify the inhibition constant of gefitinib (see $\mathtt{K_I}$ in section A.6).
- PIK3CA: PIK3CA mutation effect is implemented as an increase in PIK3CA kinase activity, estimated from *in vitro* data.
- BRAF: BRAF mutation has been reported to cause an increase in the kinase activity of MEK-phosphorylating RAF, resulting in an estimated 10-fold increase of activation, as estimated from the two most common mutations V599E and K600E. This mutation effect was implemented with such increase on the MEK activation rate in our model.
- KRAS: KRAS mutations have been reported to impair 97-99% of the GTPase activity of RAS, keeping it into a constitutively active form. As such, this mutation effect has been implemented with such reduction impact on the RAS deactivation rate.

- MET: MET Exon 14 deletion impacts the protein internalization by decreasing its degradation rate. As such, MET mutation effect was modeled as a decrease in the MET internalization and degradation rate. In addition, as the mean MET gene copy number associated with this mutation is 3.7 in stage II to IV patients, a 3.7 multiplicative factor was implemented on the total MET concentration upon MET mutation.

## A.4 EGFR signaling pathway

The objective of this submodel is to represent mechanistically EGFR (epidermal growth factor (EGF) receptor) -one receptor tyrosine kinase, RTK-signaling pathway at the molecular level, as the main pathway driving cell proliferation and survival in cancer. Based on the mutation present, and the treatment received, it will provide a cell proliferation signal and cell death inhibition signal to the Tumor Growth submodel. It also includes MET-related signaling pathways, as MET plays a critical role in resistance to EGFR inhibitor.

The submodel implements signal transduction from EGFR (EGF-dependent and independent signaling) and activation of downstream mitogen-activated protein kinase (MAPK) and phosphatidylinositol 3-kinase/protein kinase B (PI3K/AKT) pathways. ERK (extracellular signal regulated kinase) and AKT (protein kinase B) are considered as surrogate of MAPK / PI3K/AKT pathways activation status respectively.

### A.4.1 Receptor tyrosine kinase activation

This includes two parallel reactions:

- EGFR activation by EGF
- MET activation by HGF

Each RTK activation is implemented as a bidirectional reaction, following it own mass constant rate (see below), dependent, for the forward reaction, of the ligand concentration. Activation constant of MET was assumed to be of the same order of magnitude as EGFR.

$$[EGFR_{Active}] + [EGFR_{Inactive}] = Constant$$

$$[EGFR_{Active}] = k_{Fwd} * [EGF] * [EGFR_{Inactive}]$$

$$[EGFR_{Inactive}] = k_{Bwd} * [EGFR_{Active}]$$

### A.4.2 MAPK pathway activation

This includes four successive reactions:

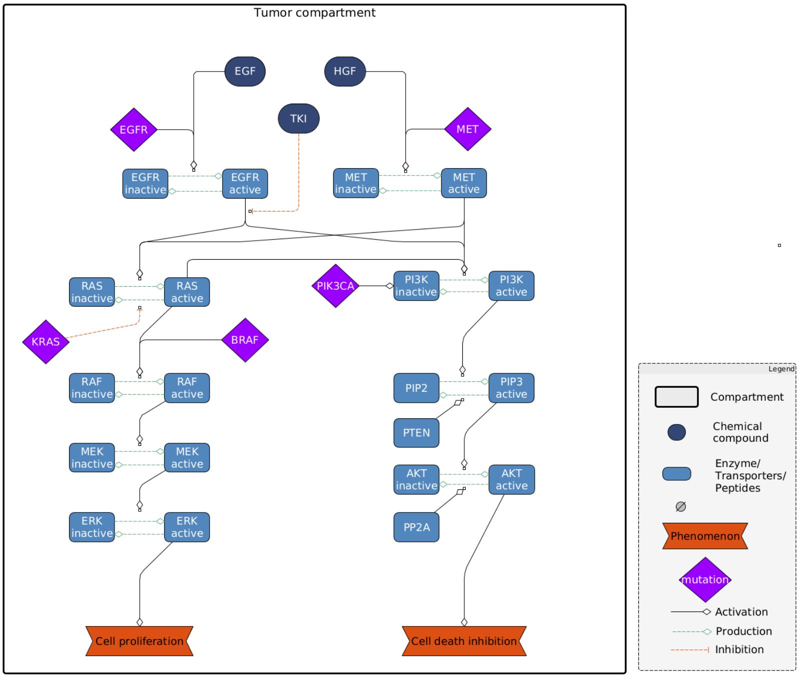


**Supplementary Figure A2** Graphical representation of the computational model of EGFR signaling pathway.

- RAS activation by EGFR and MET
- RAF activation by RAS
- MEK activation by RAF
- ERK activation by MEK

Each protein activation is implemented as a bidirectional reaction, following it own mass constant rate, dependent, for the forward reaction, of the concentration of the activated protein of the previous step (*e.g.* the forward rate of activation of RAF will depend of activated RAS concentration - see EGFR activation).

The cell proliferation signal resulting from this pathway is implemented as the ratio of the concentration of activated ERK over total ERK concentration.

### A.4.3 PI3K/AKT pathway activation

This includes three successive reactions:

- PI3K activation by EGFR, MET and RAS
- PIP2 phosphorylation into PIP3 by PI3K
- AKT activation by PIP3

Each protein/compound activation is implemented as a bidirectional reaction, following it own mass constant rate, dependent, for the forward reaction, of the concentration of the activated protein/compound of the previous step (*e.g.* the forward rate of AKT activation will depend of activated PIP3 concentration - see EGFR activation). The dephosphorylation rate of PIP3 includes two components, one corresponding to PTEN activity (and proportional to PTEN concentration), one corresponding to PIP2/PIP3 concentration homeostasis (based on our hypothesis of constant concentration of all compounds).

The cell death inhibition signal resulting from this pathway is implemented as the ratio of the concentration of activated AKT over total AKT concentration.

## A.5 Clinical submodel

The clinical submodel aims at:

- Accounting for the characteristics of the tumor in terms of size and associated clinical stage following the TNM (standing for tumor (T), nodal (N) and metastasis (M) status) Classification of Malignant Tumors.
- Representing the clinical endpoint we chose to implemente in the model: time to progression (TTP).

### A.5.1 Tumor stages - TNM classification

The clinical stage of each patient is determined through a descriptor `initialTNM` whose distribution was estimated from literature. Associated parameters encoding for the N category (`nodalStatus` parameter) and the M category (`metastasisStatus` parameter) are directly deduced from this former descriptor. These last two categories (N and M) are mainly used in the model when generating virtual population, to take into account the correlation with mutational burden.

The initial T category of an *in silico* patient impacts its initial tumor size. Each T category is defined by a range of tumor diameter. We arbitrarily set the maximal initial tumor size in the T4 category at 10 cm, and assumed that tumor size distribution within each category was uniform. The uniform distribution of tumor diameter for each stage in our model are then the following ones:

- Between 0 and 3 cm (diameter) for T1
- Between 3 and 5 cm for T2
- Between 5 and 7 cm for T3
- Between 7 and 10 cm for T4

Note that for initial T categories, subcategories (*e.g.* T1a, T1b) were not implemented as their respective abundance was not available in the reference data used to build the model.

Throughout the simulation, the T category of an *in silico* patient tumor may change depending on the evolution of the tumor size. The T category, implemented with `tumorCategory` parameter, can then be computed dynamically based on the value of `tumorRadius` :

- For `tumorRadius` = 0, T category is T0.
- For 0 < `tumorRadius` ≤ 0.5, T category is T1a.
- For 0.5 < `tumorRadius` ≤ 1, T category is T1b.
- For 1 < `tumorRadius` ≤ 1.5, T category is T1c.
- For 1.5 < `tumorRadius` ≤ 2, T category is T2a.
- For 2 < `tumorRadius` ≤ 2.5, T category is T2b.
- For 2.5 < `tumorRadius` ≤ 3.5, T category is T3.
- For `tumorRadius` > 3.5, T category is T4.

During the simulation, the clinical stage of an *in silico* patient, implemented as `clinicalStage` parameter, is then continuously inferred from tumor T, N and M category parameters introduced above.

### A.5.2 Time to progression (TTP)

We chose to implement the time to progression (TTP) as the clinical endpoint or our model. It is defined as the time elapsed between treatment initiation and tumor progression. TTP is increasingly more often used in clinical trial designs because of feasibility issues (smaller sample sizes and shorter follow-up).

Based on this definition, TTP was easily implemented in our model as the time elapsed between treatment initiation and the time at which the tumor growth rate turns positive again. It should be noted that, because tumor progression is only assessed during follow-up visits in real life setting, the simulated TTP is expected to be lower than the observed TTP.

Three estimates of TTP are modeled to take into account various factors impacting TTP measurement in clinical practice:

- **Absolute TTP**: The first TTP estimate is named the "absolute" time to progression and follows the exact definition of TTP. It thus consists in the time difference between the time of treatment initiation and the first time at which the derivative of the overall tumor cell number becomes positive. It is implemented as the `absoluteTimeToProgression` parameter.
- **Measurable TTP**: The second TTP estimate is named the "measurable" time to progression, and takes into account the measure uncertainty of imaging techniques. Indeed, typical imagery techniques such as computed tomography (CT) scan have a spatial resolution that does not allow to determine if the tumor size has increased if the increase is smaller than image resolution. We considered a typical resolution of 1 mm from CT recommendations implemented with `radialCTresolution`. parameter. The measureable TTP

is thus defined as the time elapsed between treatment initiation and the time when the tumor radius has increased by at least the the spatial resolution size. It is implemented as the `measureableTimeToProgression` parameter.
- **Clinical TTP**: The third TTP estimate is named the "clinical" time to progression, and takes into account the clinical definition of progression as defined in RECIST 1.1 guidelines. The clinical TTP is thus defined as the time elapsed between treatment initiation and the the time when the tumor radius has increased by at least 20% and at least 2.5 mm, both from the minimal size reached after treatment initiation. It is implemented as the `timeToClinicalProgression` parameter.

Based on these three definitions, `timeToClinicalProgression` should most closely reproduce the TTP reported in clinical studies. TTP will thus be calibrated on `timeToClinicalProgression`. The `absoluteTimeToProgression` and the `measureableTimeToProgression` will not be used in the calibration process but are interesting for analysis purposes as they bear information not easily measurable in clinical practice.

## A.6 Gefitinib treatment

This submodel aims is to represent pharmacokinetic and pharmacodynamic features of the first-generation EGFR-TKI drug gefitinib.

Pharmacokinetics describes how the body affects drug exposure through absorption, distribution, metabolism and excretion of the drug. Drug exposure is typically monitored in plasma, but sometimes also in the tissue where the compound exerts its pharmacodynamic effect. Pharmacodynamics describes the pharmacologic effect of the administered drug.

### A.6.1 Steady-state plasma exposure profile

We model the evolution in time of the total plasma concentration of gefitinib following daily oral administration with a 'fit-for-purpose' one-compartment linear pharmacokinetic model. Linear modeling, often associated with a one-compartment representation, is supported by preclinical studies, phase I studies and population PK models of gefitinib and is implemented here as first choice in order to reproduce plasma exposure of the compounds. The top-down approach represented by a non-physiologically-based pharmacokinetic model is the simplest way to reproduce plasma PK properties and the associated inter-patient variability, despite the fact that it does not allow a mechanistic representation of all the phenomena that influence plasma concentration. Nevertheless, the impact of phenomena related to the ADME (*i.e.* administration, distribution, metabolism and excretion) processes can still be taken into account by adjusting the kinetic parameters of the PK model.

The steady-state plasma exposure profile of the parent drug is determined by the equilibration between the rates of two reactions, one representing drug

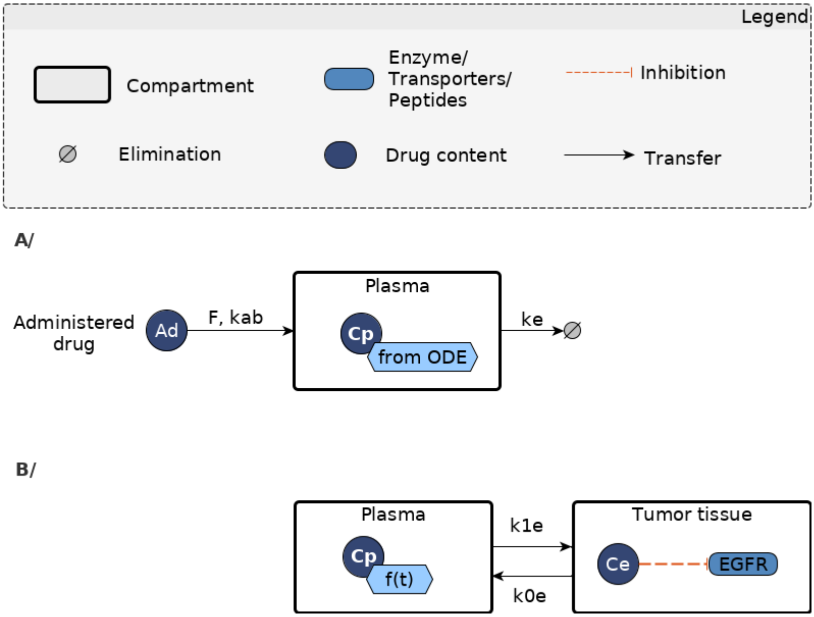


**Supplementary Figure A3** Schematic representation of the submodel structure. The goal is to reproduce plasma and tumor tissue drug concentrations (respectively Cp and Ce) as a function of time. A/ Cp is described by a one-compartment PK model, where the bioavailable fraction (F) of the administered drug is absorbed with an absorption rate kab. The drug is eliminated from plasma at an elimination rate ke. B/ Cp is input into the tumor tissue compartment, where the drug concentration Ce varies in time according to the transfer rates k1e and k0e respectively from and to the plasma compartment. The inhibition of EGFR in the tumor tissue depends on Ce. Cp can be modeled by solving a system of ordinary differential equations (ODEs) (as done in A/) but it is computationally expensive. Instead, an analytical function (called the forcing function, f(t)) that uses the same parameters (F, kab and ke) is adopted to reproduce Cp at a low computational cost and is fitted to observed plasma concentration-time data. The forcing function is input into the tumor tissue compartment. Note that the forcing function is only a function of time (f(t)) and that Ce does not affect Cp under the assumption that the tumor tissue compartment is sufficiently small.

absorption and the other one representing drug elimination. Both are reactions with first-order kinetics determining respectively absorption of the parent drug after oral administration to the plasma compartment and disappearance from it, so that:

$$C_p = \frac{A_p}{Vd/F}$$

$$\frac{dA_p}{dt} = Fk_{ab}A_d - k_eA_p$$

$$k_e = \frac{Cl/F}{Vd/F}$$

where:

- $\mathtt{C_p}$ is the total (bound + unbound) parent drug concentration in the plasma compartment
- $\mathtt{A_p}$ is the total amount of parent drug in the plasma compartment
- $\mathtt{A_d}$ is the total amount of administered drug
- F is the bioavailability
- $\mathtt{k_{ab}}$ is the first-order absorption rate constant
- $\mathtt{k_e}$ is the first-order elimination rate constant
- Cl/F is the clearance
- Vd/F is the apparent volume of distribution

and parameters $\mathtt{k_{ab}}$, Cl/F and Vd/F scale with body weight according to the formula:

$$parameterValue = refParameterValue \left( \frac{bodyWeight}{refBodyWeight} \right)^{allometricExponent}$$

with allometricExponent equal to $-0.25$ for $\mathtt{k_{ab}}$, 0.75 for Cl/F and 1 for Vd/F and refBodyWeight of 70 kg.

Additionally, the allometrically scaled parameters $Cl/F$ and $Vd/F$ both vary with age. $Cl/F$ also varies with serum $\alpha$1-acid glycoprotein concentration (mg/dL) and concomitant use of CYP3A4 inducers. The mathematical formulation of the dependency of $Cl/F$ and $Vd/F$ from the listed covariates is described in a published population pharmacokinetic model of oral gefitinib in NSCLC patients.

### A.6.2 Drug exposure in tumor tissue

The tumor tissue compartment is the compartment where the drug exerts its pharmacological effect. The steady-state total tumor tissue concentration of the parent drug is determined by the equilibration between the rates of two reactions, one representing drug entering the tumor tissue compartment from the plasma compartment and the other one representing drug elimination from the tumor tissue compartment. Both are reactions with first-order kinetics, so that:

$$\frac{dC_e}{dt} = k_{1e}C_p - k_{0e}C_e$$

where

- $\mathtt{C_p}$ is the total (bound + unbound) parent drug concentration in the plasma compartment
- Ce is the total (bound + unbound) parent drug concentration in the tumor tissue compartment
- $\mathtt{k_{1e}}$ is the first-order rate constant for transferring the drug from the plasma compartment to the tumor tissue compartment

- $k_{0e}$ is the first-order rate constant for drug elimination from the tumor tissue compartment

### A.6.3 EGFR inhibition in tumor tissue

Gefitinib is a reversible inhibitor of EGFR by competing with ATP at the intracellular kinase domain, inhibiting its autophosphorylation and, thus, attenuating the activation of intracellular signaling cascades.

We model the competitive inhibition of EGFR autophosphorylation in the tumor tissue following classical Michaelis-Menten equations for competitive inhibition:

$$pEGFR = \frac{[EGFR][ATP]}{K_M(1 + fuCe/K_I) + [ATP]}$$

where:

- EGFR is the activated (non phosphoriylated) EGFR isoform
- ATP is the ATP
- $K_M$ is the ATP Michaelis-Menten constant relative to a given EGFR isoform
- Ce is the total (bound + unbound) parent drug concentration in the tumor tissue compartment
- $K_I$ is the inhibition constant
- fu is the unbound fraction of the parent drug in the tumor tissue

Derivation of unpublished inhibitory constant values

We model the following EGFR isoforms:

- mutant L858R
- mutant Del19
- mutant A763_Y764insFQEA (exon 20 insertion)
- mutant harboring a generic resistant exon 20 insertion
- double mutant L858R/T790M
- double mutant Del19/T790M

$K_i$, the inhibition (or inhibitory) constant represents the affinity of the inhibitor for the enzyme (the lower the value, the higher the affinity). $K_i$ values as experimentally estimated by biochemical assays are not available for all the aforementioned mutants. In absence of an experimental value, we derive it from relevant $IC$ 50 data about EGFR autophosphorylation inhibition, according to the Cheng-Prusoff relationship:

$$IC_{50} = K_i(1 + \frac{[ATP]}{K_M})$$

# Supplementary material B – References

A thorough review of more than 250 scientific papers was performed to develop the ISELA model. The complete list is provided below.

## References


Adi YA, Adi-Kusumo F, Aryati L, et al (2018) A dynamic model of PI3K/AKT pathways in acute myeloid leukemia. J Appl Math 2018:1–9

Akincilar SC, Unal B, Tergaonkar V (2016) Reactivation of telomerase in cancer. Cell Mol Life Sci 73(8):1659–1670

Amin MB, Edge S, Greene FL, et al (eds) (2016) AJCC Cancer Staging Manual, 8th edn. Springer International Publishing, Cham, Switzerland

Aoki K, Yamada M, Kunida K, et al (2011) Processive phosphorylation of ERK MAP kinase in mammalian cells. Proc Natl Acad Sci U S A 108(31):12,675–12,680

Arcila ME, Nafa K, Chaft JE, et al (2013) EGFR exon 20 insertion mutations in lung adenocarcinomas: prevalence, molecular heterogeneity, and clinicopathologic characteristics. Mol Cancer Ther 12(2):220–229

Arcila ME, Drilon A, Sylvester BE, et al (2015) MAP2K1 (MEK1) mutations define a distinct subset of lung adenocarcinoma associated with smoking. Clin Cancer Res 21(8):1935–1943

Arienti C, Pignatta S, Tesei A (2019) Epidermal growth factor receptor family and its role in gastric cancer. Front Oncol 9:1308

Arteaga CL, Engelman JA (2014) ERBB receptors: from oncogene discovery to basic science to mechanism-based cancer therapeutics. Cancer Cell 25(3):282–303

Asahina H, Yamazaki K, Kinoshita I, et al (2006) A phase II trial of gefitinib as first-line therapy for advanced non-small cell lung cancer with epidermal growth factor receptor mutations. Br J Cancer 95(8):998–1004

Baltschukat S, Engstler BS, Huang A, et al (2019) Capmatinib (INC280) is active against models of non-small cell lung cancer and other cancer types with defined mechanisms of MET activation. Clin Cancer Res 25(10):3164–3175

Bertuzzi A, Conte F, Mingrone G, et al (2016) Insulin signaling in insulin resistance states and cancer: A modeling analysis. PLoS One 11(5):e0154,415

Bethune G, Bethune D, Ridgway N, et al (2010) Epidermal growth factor receptor (egfr) in lung cancer: an overview and update. J Thorac Dis 2(1):48–51

Bigot F, Boudou-Rouquette P, Arrondeau J, et al (2017) Erlotinib pharmacokinetics: a critical parameter influencing acute toxicity in elderly patients over 75 years-old. Invest New Drugs 35(2):242–246

Binnewies M, Roberts EW, Kersten K, et al (2018) Understanding the tumor immune microenvironment (TIME) for effective therapy. Nat Med 24(5):541–550

Blumenthal GM, Karuri SW, Zhang H, et al (2015) Overall response rate, progression-free survival, and overall survival with targeted and standard therapies in advanced non-small-cell lung cancer: US food and drug administration trial-level and patient-level analyses. J Clin Oncol 33(9):1008–1014

Bradley SH, Kennedy MPT, Neal RD (2019) Recognising lung cancer in primary care. Adv Ther 36(1):19–30

Brown RS, Leung JY, Kison PV, et al (1999) Glucose transporters and fdg uptake in untreated primary human non-small cell lung cancer. J Nucl Med 40(4):556–65

Brzeziński J, Lewiński A (1998) Increased plasma concentration of epidermal growth factor in female patients with non-toxic nodular goitre. Eur J Endocrinol 138(4):388–393

Bull JA, Mech F, Quaiser T, et al (2020) Mathematical modelling reveals cellular dynamics within tumour spheroids. PLoS Comput Biol 16(8):e1007,961

Burlingham BT, Widlanski TS (2003) An intuitive look at the relationship of ki and IC50: A more general use for the dixon plot. J Chem Educ 80(2):214

Campillo N, Falcones B, Otero J, et al (2019) Differential oxygenation in tumor microenvironment modulates macrophage and cancer cell crosstalk: Novel experimental setting and proof of concept. Front Oncol 9:43

Cappuzzo F, Marchetti A, Skokan M, et al (2009) Increased MET gene copy number negatively affects survival of surgically resected non-small-cell lung cancer patients. J Clin Oncol 27(10):1667–1674

Carey KD, Garton AJ, Romero MS, et al (2006) Kinetic analysis of epidermal growth factor receptor somatic mutant proteins shows increased sensitivity to the epidermal growth factor receptor tyrosine kinase inhibitor, erlotinib. Cancer Res 66(16):8163–8171

Carmona-Fontaine C, Bucci V, Akkari L, et al (2013) Emergence of spatial structure in the tumor microenvironment due to the warburg effect. Proc Natl Acad Sci U S A 110(48):19,402–19,407

Carrière A, Cargnello M, Julien LA, et al (2008) Oncogenic MAPK signaling stimulates mTORC1 activity by promoting RSK-mediated raptor phosphorylation. Curr Biol 18(17):1269–1277

Castellano E, Downward J (2011) RAS interaction with PI3K: More than just another effector pathway. Genes Cancer 2(3):261–274

Chang YS, Choi CM, Lee JC (2016) Mechanisms of epidermal growth factor receptor tyrosine kinase inhibitor resistance and strategies to overcome resistance in lung adenocarcinoma. Tuberc Respir Dis (Seoul) 79(4):248–256

Chen YR, Fu YN, Lin CH, et al (2006) Distinctive activation patterns in constitutively active and gefitinib-sensitive EGFR mutants. Oncogene 25(8):1205–1215

Choi K, Medley JK, König M, et al (2018) Tellurium: An extensible python-based modeling environment for systems and synthetic biology. Biosystems 171:74–79. https://doi.org/https://doi.org/10.1016/j.biosystems.2018.07.006, URL https://www.sciencedirect.com/science/article/pii/S0303264718301254

Coche E (2016) Evaluation of lung tumor response to therapy: Current and emerging techniques. Diagn Interv Imaging 97(10):1053–1065

Collins DM, Conlon NT, Kannan S, et al (2019) Preclinical characteristics of the irreversible pan-HER kinase inhibitor neratinib compared with lapatinib: Implications for the treatment of HER2-positive and HER2-mutated breast cancer. Cancers (Basel) 11(6):737

Colotta F, Allavena P, Sica A, et al (2009) Cancer-related inflammation, the seventh hallmark of cancer: links to genetic instability. Carcinogenesis 30(7):1073–1081

Cross DAE, Ashton SE, Ghiorghiu S, et al (2014) AZD9291, an irreversible EGFR TKI, overcomes T790M-mediated resistance to EGFR inhibitors in lung cancer. Cancer Discov 4(9):1046–1061

Curtin L, Hawkins-Daarud A, Porter AB, et al (2020) A mechanistic investigation into ischemia-driven distal recurrence of glioblastoma

Curto M, Cole BK, Lallemand D, et al (2007) Contact-dependent inhibition of EGFR signaling by Nf2/Merlin. J Cell Biol 177(5):893–903

Dagogo-Jack I, Shaw AT (2018) Tumour heterogeneity and resistance to cancer therapies. Nat Rev Clin Oncol 15(2):81–94

Dal Maso A, Lorenzi M, Roca E, et al (2020) Clinical features and progression pattern of acquired T790M-positive compared with T790M-negative EGFR mutant non-small-cell lung cancer: Catching tumor and clinical heterogeneity over time through liquid biopsy. Clin Lung Cancer 21(1):1–14.e3

Davies H, Bignell GR, Cox C, et al (2002) Mutations of the BRAF gene in human cancer. Nature 417(6892):949–954

Davis A, Gao R, Navin N (2017) Tumor evolution: Linear, branching, neutral or punctuated? Biochim Biophys Acta 1867(2):151–161

De Wever W, Coolen J, Verschakelen JA (2011) Imaging techniques in lung cancer. Breathe (Sheff) 7(4):338–346

Deeks ED, Keating GM (2018) Afatinib in advanced NSCLC: a profile of its use. Drugs Ther Perspect 34(3):89–98

Del Re M, Crucitta S, Gianfilippo G, et al (2019) Understanding the mechanisms of resistance in EGFR-positive NSCLC: From tissue to liquid biopsy to guide treatment strategy. Int J Mol Sci 20(16):3951

Deprez M, Zaragosi LE, Truchi M, et al (2019) A single-cell atlas of the human healthy airways

Domagala-Kulawik J (2015) The role of the immune system in non-small cell lung carcinoma and potential for therapeutic intervention. Transl Lung Cancer Res 4(2):177–190

Dong Y, Tu R, Liu H, et al (2020) Regulation of cancer cell metabolism: oncogenic MYC in the driver's seat. Signal Transduct Target Ther 5(1):124

Donninger H, Vos MD, Clark GJ (2007) The RASSF1A tumor suppressor. J Cell Sci 120(Pt 18):3163–3172

Duda P, Akula SM, Abrams SL, et al (2020) Targeting GSK3 and associated signaling pathways involved in cancer. Cells 9(5):1110

Eck MJ, Yun CH (2010) Structural and mechanistic underpinnings of the differential drug sensitivity of EGFR mutations in non-small cell lung cancer. Biochim Biophys Acta 1804(3):559–566

Eigenmann MJ, Frances N, Hoffmann G, et al (2016) Combining nonclinical experiments with translational PKPD modeling to differentiate erlotinib and gefitinib. Mol Cancer Ther 15(12):3110–3119

Eigenmann MJ, Frances N, Lavé T, et al (2017) PKPD modeling of acquired resistance to anti-cancer drug treatment. J Pharmacokinet Pharmacodyn 44(6):617–630

Eisenhauer EA, Therasse P, Bogaerts J, et al (2009) New response evaluation criteria in solid tumours: revised RECIST guideline (version 1.1). Eur J Cancer 45(2):228–247

Ekert JE, Johnson K, Strake B, et al (2014) Three-dimensional lung tumor microenvironment modulates therapeutic compound responsiveness in vitro–implication for drug development. PLoS One 9(3):e92,248

Emery CM, Monaco KA, Wang P, et al (2017) BRAF-inhibitor associated MEK mutations increase RAF-dependent and -independent enzymatic activity. Mol Cancer Res 15(10):1431–1444

Emmerson J, Brown JM (2021) Understanding survival analysis in clinical trials. Clin Oncol (R Coll Radiol) 33(1):12–14

Endo H, Okami J, Okuyama H, et al (2013) Spheroid culture of primary lung cancer cells with neuregulin 1/HER3 pathway activation. J Thorac Oncol 8(2):131–139

van Erp NP, Gelderblom H, Guchelaar HJ (2009) Clinical pharmacokinetics of tyrosine kinase inhibitors. Cancer Treat Rev 35(8):692–706

Fang W, Huang Y, Hong S, et al (2019) EGFR exon 20 insertion mutations and response to osimertinib in non-small-cell lung cancer. BMC Cancer 19(1):595

Ferlay J, Colombet M, Soerjomataram I, et al (2018) Cancer incidence and mortality patterns in europe: Estimates for 40 countries and 25 major cancers in 2018. Eur J Cancer 103:356–387

Ferrer I, Zugazagoitia J, Herbertz S, et al (2018) KRAS-Mutant non-small cell lung cancer: From biology to therapy. Lung Cancer 124:53–64

Fontanini G, De Laurentiis M, Vignati S, et al (1998) Evaluation of epidermal growth factor-related growth factors and receptors and of neoangiogenesis in completely resected stage i-iiia non-small-cell lung cancer: amphiregulin and microvessel count are independent prognostic indicators of survival. Clin Cancer Res 4(1):241–249

Freyer JP (1988) Role of necrosis in regulating the growth saturation of multicellular spheroids. Cancer Res 48(9):2432–2439

Frohna P, Lu J, Eppler S, et al (2006) Evaluation of the absolute oral bioavailability and bioequivalence of erlotinib, an inhibitor of the epidermal growth factor receptor tyrosine kinase, in a randomized, crossover study in healthy subjects. J Clin Pharmacol 46(3):282–290

Fujiwara S, Hung M, Yamamoto-Ibusuk CM, et al (2014) The localization of HER4 intracellular domain and expression of its alternately-spliced isoforms have prognostic significance in ER+ HER2- breast cancer. Oncotarget 5(11):3919–3930

Garnett MJ, Marais R (2004) Guilty as charged: B-RAF is a human oncogene. Cancer Cell 6(4):313–319

Gazdar AF (2009) Activating and resistance mutations of EGFR in non-small-cell lung cancer: role in clinical response to EGFR tyrosine kinase inhibitors. Oncogene 28 Suppl 1(S1):S24–31

Gelatti ACZ, Drilon A, Santini FC (2019) Optimizing the sequencing of tyrosine kinase inhibitors (TKIs) in epidermal growth factor receptor (EGFR) mutation-positive non-small cell lung cancer (NSCLC). Lung Cancer 137:113–122

George B, Seals S, Aban I (2014) Survival analysis and regression models. J Nucl Cardiol 21(4):686–694

Ghosh R, Narasanna A, Wang SE, et al (2011) Trastuzumab has preferential activity against breast cancers driven by HER2 homodimers. Cancer Res 71(5):1871–1882

Giroux V, Rustgi AK (2017) Metaplasia: tissue injury adaptation and a precursor to the dysplasia-cancer sequence. Nat Rev Cancer 17(10):594–604

Glotzer OS, Fabian T, Chandra A, et al (2013) Non-small cell lung cancer therapy: safety and efficacy in the elderly. Drug Healthc Patient Saf 5:113–121

Gonzalez H, Hagerling C, Werb Z (2018) Roles of the immune system in cancer: from tumor initiation to metastatic progression. Genes Dev 32(19-20):1267–1284

Gould MK, Kuschner WG, Rydzak CE, et al (2003) Test performance of positron emission tomography and computed tomography for mediastinal staging in patients with non-small-cell lung cancer: a meta-analysis. Ann Intern Med 139(11):879–892

Grassberger C, McClatchy D3rd, Geng C, et al (2019) Patient-specific tumor growth trajectories determine persistent and resistant cancer cell populations during treatment with targeted therapies. Cancer Res 79(14):3776–3788

Greaves M, Maley CC (2012) Clonal evolution in cancer. Nature 481(7381):306–313

Gribble FM, Loussouarn G, Tucker SJ, et al (2000) A novel method for measurement of submembrane ATP concentration. J Biol Chem 275(39):30,046–30,049

Gridelli C, Rossi A, Carbone DP, et al (2015) Non-small-cell lung cancer. Nat Rev Dis Primers 1:15,009

Grkovski M, Schwartz J, Rimner A, et al (2016) Reproducibility of 18f-fluoromisonidazole intratumour distribution in non-small cell lung cancer. EJNMMI Res 6(1):79

Grosse A, Grosse C, Rechsteiner M, et al (2019) Analysis of the frequency of oncogenic driver mutations and correlation with clinicopathological characteristics in patients with lung adenocarcinoma from northeastern switzerland. Diagn Pathol 14(1):18

Guha U, Chaerkady R, Marimuthu A, et al (2008) Comparisons of tyrosine phosphorylated proteins in cells expressing lung cancer-specific alleles of EGFR and KRAS. Proc Natl Acad Sci U S A 105(37):14,112–14,117

Guo G, Gong K, Wohlfeld B, et al (2015) Ligand-independent EGFR signaling. Cancer Res 75(17):3436–3441

Halliday PR, Blakely CM, Bivona TG (2019) Emerging targeted therapies for the treatment of non-small cell lung cancer. Curr Oncol Rep 21(3):21

Hanahan D, Weinberg RA (2000) The hallmarks of cancer. Cell 100(1):57–70

Hanahan D, Weinberg RA (2011) Hallmarks of cancer: the next generation. Cell 144(5):646–674

Harrison PT, Vyse S, Huang PH (2020) Rare epidermal growth factor receptor (EGFR) mutations in non-small cell lung cancer. Semin Cancer Biol 61:167–179

Hartl D, Tirouvanziam R, Laval J, et al (2018) Innate immunity of the lung: From basic mechanisms to translational medicine. J Innate Immun 10(5-6):487–501

Heldin CH, Rubin K, Pietras K, et al (2004) High interstitial fluid pressure - an obstacle in cancer therapy. Nat Rev Cancer 4(10):806–813

Helfrich BA, Raben D, Varella-Garcia M, et al (2006) Antitumor activity of the epidermal growth factor receptor (EGFR) tyrosine kinase inhibitor gefitinib (ZD1839, iressa) in non-small cell lung cancer cell lines correlates with gene copy number and EGFR mutations but not EGFR protein levels. Clin Cancer Res 12(23):7117–7125

Hermida MA, Dinesh Kumar J, Leslie NR (2017) GSK3 and its interactions with the PI3K/AKT/mTOR signalling network. Adv Biol Regul 65:5–15

Hidalgo M, Siu LL, Nemunaitis J, et al (2001) Phase I and pharmacologic study of OSI-774, an epidermal growth factor receptor tyrosine kinase inhibitor, in patients with advanced solid malignancies. J Clin Oncol 19(13):3267–3279

Hirschey MD, DeBerardinis RJ, Diehl AME, et al (2015) Dysregulated metabolism contributes to oncogenesis. Semin Cancer Biol 35 Suppl:S129–S150

Hsu PC, Chang JWC, Wang CC, et al (2019) Oral vinorelbine plus cisplatin with concomitant radiotherapy as induction therapy for stage III non-small cell lung cancer: Results of a single-arm prospective cohort study. Thorac Cancer 10(8):1683–1691

Hua H, Kong Q, Zhang H, et al (2019) Targeting mTOR for cancer therapy. J Hematol Oncol 12(1):71

Huang YH, Hsu KH, Tseng JS, et al (2018) The association of acquired T790M mutation with clinical characteristics after resistance to first-line epidermal growth factor receptor tyrosine kinase inhibitor in lung adenocarcinoma. Cancer Res Treat 50(4):1294–1303

Hunter JC, Manandhar A, Carrasco MA, et al (2015) Biochemical and structural analysis of common cancer-associated KRAS mutations. Mol Cancer Res 13(9):1325–1335

Ikenoue T, Hikiba Y, Kanai F, et al (2003) Functional analysis of mutations within the kinase activation segment of b-raf in human colorectal tumors. Cancer Res 63(23):8132–8137

Islam KMM, Jiang X, Anggondowati T, et al (2015) Comorbidity and survival in lung cancer patients. Cancer Epidemiol Biomarkers Prev 24(7):1079–1085

Jackman DM, Yeap BY, Sequist LV, et al (2006) Exon 19 deletion mutations of epidermal growth factor receptor are associated with prolonged survival in non-small cell lung cancer patients treated with gefitinib or erlotinib. Clin Cancer Res 12(13):3908–3914

Jagiella N, Müller B, Müller M, et al (2016) Inferring growth control mechanisms in growing multi-cellular spheroids of NSCLC cells from spatial-temporal image data. PLoS Comput Biol 12(2):e1004,412

Jamal-Hanjani M, Wilson GA, McGranahan N, et al (2017) Tracking the evolution of non-small-cell lung cancer. N Engl J Med 376(22):2109–2121

Jassem J (2019) Adjuvant EGFR tyrosine kinase inhibitors in EGFR-mutant non-small cell lung cancer: still an investigational approach. Transl Lung Cancer Res 8(Suppl 4):S387–S390

Jia Q, Wu W, Wang Y, et al (2018) Local mutational diversity drives intratumoral immune heterogeneity in non-small cell lung cancer. Nat Commun 9(1):5361

Johnson JL, Pillai S, Chellappan SP (2012) Genetic and biochemical alterations in non-small cell lung cancer. Biochem Res Int 2012:940,405

Johnson JR, Cohen M, Sridhara R, et al (2005) Approval summary for erlotinib for treatment of patients with locally advanced or metastatic non-small cell lung cancer after failure of at least one prior chemotherapy regimen. Clin Cancer Res 11(18):6414–6421

Jones RG, Thompson CB (2009) Tumor suppressors and cell metabolism: a recipe for cancer growth. Genes Dev 23(5):537–548

Jorge SE, Lucena-Araujo AR, Yasuda H, et al (2018) EGFR exon 20 insertion mutations display sensitivity to hsp90 inhibition in preclinical models and lung adenocarcinomas. Clin Cancer Res 24(24):6548–6555

Jorge SEDC, Kobayashi SS, Costa DB (2014) Epidermal growth factor receptor (EGFR) mutations in lung cancer: preclinical and clinical data. Braz J Med Biol Res 47(11):929–939

Joyce JA (2005) Therapeutic targeting of the tumor microenvironment. Cancer Cell 7(6):513–520

Junttila MR, de Sauvage FJ (2013) Influence of tumour micro-environment heterogeneity on therapeutic response. Nature 501(7467):346–354

Kalluri R, Weinberg RA (2009) The basics of epithelial-mesenchymal transition. J Clin Invest 119(6):1420–1428

Kang HN, Choi JW, Shim HS, et al (2018) Establishment of a platform of non-small-cell lung cancer patient-derived xenografts with clinical and genomic annotation. Lung Cancer 124:168–178

Kato S, Okamura R, Mareboina M, et al (2019) Revisiting epidermal growth factor receptor (EGFR) amplification as a target for anti-EGFR therapy: Analysis of cell-free circulating tumor DNA in patients with advanced malignancies. JCO Precis Oncol 3(3):1–14

Katsuya Y, Fujiwara Y, Sunami K, et al (2015) Comparison of the pharmacokinetics of erlotinib administered in complete fasting and 2 h after a meal in patients with lung cancer. Cancer Chemother Pharmacol 76(1):125–132

Kawata T, Higashimori M, Itoh Y, et al (2019) Gefitinib exposure and occurrence of interstitial lung disease in japanese patients with non-small-cell lung cancer. Cancer Chemother Pharmacol 83(5):849–858

Kelada OJ, Rockwell S, Zheng MQ, et al (2017) Quantification of tumor hypoxic fractions using positron emission tomography with [18f]fluoromisonidazole ([18F]FMISO) kinetic analysis and invasive oxygen measurements. Mol Imaging Biol 19(6):893–902

Khunger A, Khunger M, Velcheti V (2018) Dabrafenib in combination with trametinib in the treatment of patients with BRAF v600-positive advanced or metastatic non-small cell lung cancer: clinical evidence and experience. Ther Adv Respir Dis 12:1753466618767,611

Kim Y, Apetri M, Luo B, et al (2015) Differential effects of tyrosine kinase inhibitors on normal and oncogenic EGFR signaling and downstream effectors. Mol Cancer Res 13(4):765–774

Kluge A, Dabir S, Vlassenbroeck I, et al (2011) Protein inhibitor of activated STAT3 expression in lung cancer. Mol Oncol 5(3):256–264

Kobayashi Y, Mitsudomi T (2016) Not all epidermal growth factor receptor mutations in lung cancer are created equal: Perspectives for individualized treatment strategy. Cancer Sci 107(9):1179–1186

Kucharczuk R, Alex G, Vozniak M (2018) Drug-drug interactions, safety, and pharmacokinetics of EGFR tyrosine kinase inhibitors for the treatment of non–small cell lung cancer. J Adv Pract Oncol 9(2)

Kumar A, Petri ET, Halmos B, et al (2008) Structure and clinical relevance of the epidermal growth factor receptor in human cancer. J Clin Oncol 26(10):1742–1751

Lantz PM, Mendez D, Philbert MA (2013) Radon, smoking, and lung cancer: the need to refocus radon control policy. Am J Public Health 103(3):443–447

Laplante M, Sabatini DM (2012) mTOR signaling in growth control and disease. Cell 149(2):274–293

Lavacchi D, Mazzoni F, Giaccone G (2019) Clinical evaluation of dacomitinib for the treatment of metastatic non-small cell lung cancer (NSCLC): current perspectives. Drug Des Devel Ther 13:3187–3198

Lavin Y, Kobayashi S, Leader A, et al (2017) Innate immune landscape in early lung adenocarcinoma by paired single-cell analyses. Cell 169(4):750–765.e17

Lee CYF, Lin Y, Bratman SV, et al (2014) Neuregulin autocrine signaling promotes self-renewal of breast tumor-initiating cells by triggering HER2/HER3 activation. Cancer Res 74(1):341–352

Lee SH, Jeong D, Han YS, et al (2015) Pivotal role of vascular endothelial growth factor pathway in tumor angiogenesis. Ann Surg Treat Res 89(1):1–8

Leonetti A, Sharma S, Minari R, et al (2019) Resistance mechanisms to osimertinib in EGFR-mutated non-small cell lung cancer. Br J Cancer 121(9):725–737

Leung KM, Elashoff RM, Afifi AA (1997) Censoring issues in survival analysis. Annu Rev Public Health 18(1):83–104

Leventakos K, Kipp BR, Rumilla KM, et al (2016) S768I mutation in EGFR in patients with lung cancer. J Thorac Oncol 11(10):1798–1801

Lewin TD, Maini PK, Moros EG, et al (2018) The evolution of tumour composition during fractionated radiotherapy: Implications for outcome. Bull Math Biol 80(5):1207–1235

Liang H, Wang M (2020) MET oncogene in non-small cell lung cancer: Mechanism of MET dysregulation and agents targeting the HGF/c-Met axis. Onco Targets Ther 13:2491–2510

Ling J, Johnson KA, Miao Z, et al (2006) Metabolism and excretion of erlotinib, a small molecule inhibitor of epidermal growth factor receptor tyrosine kinase, in healthy male volunteers. Drug Metab Dispos 34(3):420–426

Liotta LA, Kohn EC (2001) The microenvironment of the tumour-host interface. Nature 411(6835):375–379

LoRusso PM (2016) Inhibition of the PI3K/AKT/mTOR pathway in solid tumors. J Clin Oncol 34(31):3803–3815

Lu JF, Eppler SM, Wolf J, et al (2006) Clinical pharmacokinetics of erlotinib in patients with solid tumors and exposure-safety relationship in patients with non-small cell lung cancer. Clin Pharmacol Ther 80(2):136–145

Lu X, Peled N, Greer J, et al (2017) *MET* exon 14 mutation encodes an actionable therapeutic target in lung adenocarcinoma. Cancer Res 77(16):4498–4505

Ma C, Wei S, Song Y (2011) T790M and acquired resistance of EGFR TKI: a literature review of clinical reports. J Thorac Dis 3(1):10–18

Maemondo M, Inoue A, Kobayashi K, et al (2010) Gefitinib or chemotherapy for non-small-cell lung cancer with mutated EGFR. N Engl J Med 362(25):2380–2388

Maity S, Pai KSR, Nayak Y (2020) Advances in targeting EGFR allosteric site as anti-NSCLC therapy to overcome the drug resistance. Pharmacol Rep 72(4):799–813

Manning AL, Dyson NJ (2011) pRB, a tumor suppressor with a stabilizing presence. Trends Cell Biol 21(8):433–441

Manzo A, Montanino A, Carillio G, et al (2017) Angiogenesis inhibitors in NSCLC. Int J Mol Sci 18(10)

Marusyk A, Polyak K (2010) Tumor heterogeneity: causes and consequences. Biochim Biophys Acta 1805(1):105–117

Massarelli E, Varella-Garcia M, Tang X, et al (2007) KRAS mutation is an important predictor of resistance to therapy with epidermal growth factor receptor tyrosine kinase inhibitors in non-small-cell lung cancer. Clin Cancer Res 13(10):2890–2896

Maurer U, Preiss F, Brauns-Schubert P, et al (2014) GSK-3 - at the crossroads of cell death and survival. J Cell Sci 127(Pt 7):1369–1378

McGranahan N, Swanton C (2017) Clonal heterogeneity and tumor evolution: Past, present, and the future. Cell 168(4):613–628

McKillop D, Partridge EA, Kemp JV, et al (2005) Tumor penetration of gefitinib (iressa), an epidermal growth factor receptor tyrosine kinase inhibitor. Mol Cancer Ther 4(4):641–649

McKillop D, Guy SP, Spence MP, et al (2006) Minimal contribution of desmethyl-gefitinib, the major human plasma metabolite of gefitinib, to epidermal growth factor receptor (EGFR)-mediated tumour growth inhibition. Xenobiotica 36(1):29–39

Meany HJ, Fox E, McCully C, et al (2008) The plasma and cerebrospinal fluid pharmacokinetics of erlotinib and its active metabolite (OSI-420) after intravenous administration of erlotinib in non-human primates. Cancer Chemother Pharmacol 62(3):387–392

Medina M, Wandosell F (2011) Deconstructing GSK-3: The fine regulation of its activity. Int J Alzheimers Dis 2011:479,249

Medley JK, Choi K, König M, et al (2018) Tellurium notebooks—an environment for reproducible dynamical modeling in systems biology. PLOS Computational Biology 14(6):1–24. https://doi.org/10.1371/journal.pcbi.1006220, URL https://doi.org/10.1371/journal.pcbi.1006220

Mehta G, Hsiao AY, Ingram M, et al (2012) Opportunities and challenges for use of tumor spheroids as models to test drug delivery and efficacy. J Control Release 164(2):192–204

Meisel A, Hochmair MProf (2020) Critical review of EGFR-mutated NSCLC: What we do and do not know. healthbook TIMES Onco Hema (3):20–35

Mello SS, Attardi LD (2018) Deciphering p53 signaling in tumor suppression. Curr Opin Cell Biol 51:65–72

Mendoza MC, Er EE, Blenis J (2011) The Ras-ERK and PI3K-mTOR pathways: cross-talk and compensation. Trends Biochem Sci 36(6):320–328

Meng X, Kong FMS, Yu J (2012) Implementation of hypoxia measurement into lung cancer therapy. Lung Cancer 75(2):146–150

Meyer R, D'Alessandro LA, Kar S, et al (2012) Heterogeneous kinetics of AKT signaling in individual cells are accounted for by variable protein concentration. Front Physiol 3:451

Milella M, Falcone I, Conciatori F, et al (2015) PTEN: Multiple functions in human malignant tumors. Front Oncol 5:24

Mimori K, Saito T, Niida A, et al (2018) Cancer evolution and heterogeneity. Ann Gastroenterol Surg 2(5):332–338

Minari R, Bordi P, Tiseo M (2016) Third-generation epidermal growth factor receptor-tyrosine kinase inhibitors in T790M-positive non-small cell lung cancer: review on emerged mechanisms of resistance. Transl Lung Cancer Res 5(6):695–708

Mishra R, Patel H, Alanazi S, et al (2018) HER3 signaling and targeted therapy in cancer. Oncol Rev 12(1):355

Moores SL, Chiu ML, Bushey BS, et al (2016) A novel bispecific antibody targeting EGFR and cmet is effective against EGFR inhibitor-resistant lung tumors. Cancer Res 76(13):3942–3953

Morgillo F, Della Corte CM, Fasano M, et al (2016) Mechanisms of resistance to EGFR-targeted drugs: lung cancer. ESMO Open 1(3):e000,060

Nakakuki T, Yumoto N, Naka T, et al (2008) Topological analysis of MAPK cascade for kinetic ErbB signaling. PLoS One 3(3):e1782

Nguyen KSH, Kobayashi S, Costa DB (2009) Acquired resistance to epidermal growth factor receptor tyrosine kinase inhibitors in non-small-cell lung cancers dependent on the epidermal growth factor receptor pathway. Clin Lung Cancer 10(4):281–289

Nishida N, Yano H, Nishida T, et al (2006) Angiogenesis in cancer. Vasc Health Risk Manag 2(3):213–219

Nishino M, Dahlberg SE, Cardarella S, et al (2013) Tumor volume decrease at 8 weeks is associated with longer survival in EGFR-mutant advanced non-small-cell lung cancer patients treated with EGFR TKI. J Thorac Oncol 8(8):1059–1068

Nunes AS, Barros AS, Costa EC, et al (2019) 3D tumor spheroids as in vitro models to mimic in vivo human solid tumors resistance to therapeutic drugs. Biotechnol Bioeng 116(1):206–226

Öjlert ÅK, Halvorsen AR, Nebdal D, et al (2019) The immune microenvironment in non-small cell lung cancer is predictive of prognosis after surgery. Mol Oncol 13(5):1166–1179

Okada T, Lopez-Lago M, Giancotti FG (2005) Merlin/NF-2 mediates contact inhibition of growth by suppressing recruitment of rac to the plasma membrane. J Cell Biol 171(2):361–371

Ortiz-Zapater E, Lee RW, Owen W, et al (2017) MET-EGFR dimerization in lung adenocarcinoma is dependent on EGFR mtations and altered by MET kinase inhibition. PLoS One 12(1):e0170,798

Osmani L, Askin F, Gabrielson E, et al (2018) Current WHO guidelines and the critical role of immunohistochemical markers in the subclassification of non-small cell lung carcinoma (NSCLC): Moving from targeted therapy to immunotherapy. Semin Cancer Biol 52(Pt 1):103–109

Oxnard GR, Hu Y, Mileham KF, et al (2018) Assessment of resistance mechanisms and clinical implications in patients with EGFR T790M-positive lung cancer and acquired resistance to osimertinib. JAMA Oncol 4(11):1527–1534

Pandey MK, DeGrado TR (2016) Glycogen synthase kinase-3 (GSK-3)-targeted therapy and imaging. Theranostics 6(4):571–593

Papandreou I, Krishna C, Kaper F, et al (2005) Anoxia is necessary for tumor cell toxicity caused by a low-oxygen environment. Cancer Res 65(8):3171–3178

Peckys DB, Korf U, de Jonge N (2015) Local variations of HER2 dimerization in breast cancer cells discovered by correlative fluorescence and liquid electron microscopy. Sci Adv 1(6):e1500,165

Peinado H, Olmeda D, Cano A (2007) Snail, zeb and bHLH factors in tumour progression: an alliance against the epithelial phenotype? Nat Rev Cancer 7(6):415–428

Pines G, Köstler WJ, Yarden Y (2010) Oncogenic mutant forms of EGFR: lessons in signal transduction and targets for cancer therapy. FEBS Lett 584(12):2699–2706

Postmus PE, Kerr KM, Oudkerk M, et al (2017) Early and locally advanced non-small-cell lung cancer (NSCLC): ESMO clinical practice guidelines for diagnosis, treatment and follow-up. Ann Oncol 28(suppl 4):iv1–iv21

Pugh SL (2017) Essence of survival analysis. Neurooncol Pract 4(2):77–81

Puri N, Salgia R (2008) Synergism of EGFR and c-met pathways, cross-talk and inhibition, in non-small cell lung cancer. J Carcinog 7(1):9

Quintanal-Villalonga A, Paz-Ares L, Ferrer I, et al (2016) Tyrosine kinase receptor landscape in lung cancer: Therapeutical implications. Dis Markers 2016:9214,056

Ranson M, Hammond LA, Ferry D, et al (2002) ZD1839, a selective oral epidermal growth factor receptor-tyrosine kinase inhibitor, is well tolerated and active in patients with solid, malignant tumors: results of a phase I trial. J Clin Oncol 20(9):2240–2250

Ranson M, Shaw H, Wolf J, et al (2010) A phase I dose-escalation and bioavailability study of oral and intravenous formulations of erlotinib (tarceva, OSI-774) in patients with advanced solid tumors of epithelial origin. Cancer Chemother Pharmacol 66(1):53–58

Rawlings JS, Rosler KM, Harrison DA (2004) The JAK/STAT signaling pathway. J Cell Sci 117(Pt 8):1281–1283

Reed DE, Shokat KM (2017) INPP4B and PTEN loss leads to PI-3,4-P2 accumulation and inhibition of PI3K in TNBC. Mol Cancer Res 15(6):765–775

Reguart N, Remon J (2015) Common EGFR-mutated subgroups (Del19/L858R) in advanced non-small-cell lung cancer: chasing better outcomes with tyrosine kinase inhibitors. Future Oncol 11(8):1245–1257

Ribba B, Watkin E, Tod M, et al (2011) A model of vascular tumour growth in mice combining longitudinal tumour size data with histological biomarkers. Eur J Cancer 47(3):479–490

Riely GJ, Pao W, Pham D, et al (2006) Clinical course of patients with non-small cell lung cancer and epidermal growth factor receptor exon 19 and exon 21 mutations treated with gefitinib or erlotinib. Clin Cancer Res 12(3 Pt 1):839–844

Riess JW, Gandara DR, Frampton GM, et al (2018) Diverse EGFR exon 20 insertions and co-occurring molecular alterations identified by comprehensive genomic profiling of NSCLC. J Thorac Oncol 13(10):1560–1568

Rodgers SJ, Ferguson DT, Mitchell CA, et al (2017) Regulation of PI3K effector signalling in cancer by the phosphoinositide phosphatases. Biosci Rep 37(1)

Romagosa C, Simonetti S, López-Vicente L, et al (2011) p16(ink4a) overexpression in cancer: a tumor suppressor gene associated with senescence and high-grade tumors. Oncogene 30(18):2087–2097

Román M, Baraibar I, López I, et al (2018) KRAS oncogene in non-small cell lung cancer: clinical perspectives on the treatment of an old target. Mol Cancer 17(1):33

Rotow J, Bivona TG (2017) Understanding and targeting resistance mechanisms in NSCLC. Nat Rev Cancer 17(11):637–658

Rubin GD (2015) Lung nodule and cancer detection in computed tomography screening. J Thorac Imaging 30(2):130–138

Russo A, Franchina T, Ricciardi G, et al (2019) Heterogeneous responses to epidermal growth factor receptor (EGFR) tyrosine kinase inhibitors (TKIs) in patients with uncommon EGFR mutations: New insights and future perspectives in this complex clinical scenario. Int J Mol Sci 20(6):1431

Saad ED, Katz A (2009) Progression-free survival and time to progression as primary end points in advanced breast cancer: often used, sometimes loosely defined. Ann Oncol 20(3):460–464

Saito H, Fukuhara T, Furuya N, et al (2019) Erlotinib plus bevacizumab versus erlotinib alone in patients with EGFR-positive advanced non-squamous non-small-cell lung cancer (NEJ026): interim analysis of an open-label, randomised, multicentre, phase 3 trial. Lancet Oncol 20(5):625–635

Salem A, Asselin MC, Reymen B, et al (2018) Targeting hypoxia to improve non-small cell lung cancer outcome. J Natl Cancer Inst 110(1):14–30

Samaga R, Saez-Rodriguez J, Alexopoulos LG, et al (2009) The logic of EGFR/ErbB signaling: theoretical properties and analysis of high-throughput data. PLoS Comput Biol 5(8):e1000,438

Santarpia L, Lippman SM, El-Naggar AK (2012) Targeting the MAPK-RAS-RAF signaling pathway in cancer therapy. Expert Opin Ther Targets 16(1):103–119

Santoni-Rugiu E, Melchior LC, Urbanska EM, et al (2019) Intrinsic resistance to EGFR-tyrosine kinase inhibitors in EGFR-mutant non-small cell lung cancer: Differences and similarities with acquired resistance. Cancers (Basel) 11(7):923

Scacheri CA, Scacheri PC (2015) Mutations in the noncoding genome. Curr Opin Pediatr 27(6):659–664

Scaltriti M, Baselga J (2006) The epidermal growth factor receptor pathway: a model for targeted therapy. Clin Cancer Res 12(18):5268–5272

Scheffler M, Zander T, Nogova L, et al (2013) Prognostic impact of [18f]fluorothymidine and [18F]fluoro-D-glucose baseline uptakes in patients with lung cancer treated first-line with erlotinib. PLoS One 8(1):e53,081

Schlender JF, Meyer M, Thelen K, et al (2016) Development of a whole-body physiologically based pharmacokinetic approach to assess the pharmacokinetics of drugs in elderly individuals. Clin Pharmacokinet 55(12):1573–1589

Schoeberl B, Eichler-Jonsson C, Gilles ED, et al (2002) Computational modeling of the dynamics of the MAP kinase cascade activated by surface and internalized EGF receptors. Nat Biotechnol 20(4):370–375

Schrank Z, Chhabra G, Lin L, et al (2018) Current molecular-targeted therapies in NSCLC and their mechanism of resistance. Cancers (Basel) 10(7)

Ségal-Bendirdjian E, Geli V (2019) Non-canonical roles of telomerase: Unraveling the imbroglio. Front Cell Dev Biol 7:332

Seo JS, Kim A, Shin JY, et al (2018) Comprehensive analysis of the tumor immune micro-environment in non-small cell lung cancer for efficacy of checkpoint inhibitor. Sci Rep 8(1):14,576

Seto T, Kato T, Nishio M, et al (2014) Erlotinib alone or with bevacizumab as first-line therapy in patients with advanced non-squamous non-small-cell lung cancer harbouring EGFR mutations (JO25567): an open-label, randomised, multicentre, phase 2 study. Lancet Oncol 15(11):1236–1244

Shah R, Lester JF (2020) Tyrosine kinase inhibitors for the treatment of EGFR mutation-positive non-small-cell lung cancer: A clash of the generations. Clin Lung Cancer 21(3):e216–e228

Shan F, Shao Z, Jiang S, et al (2016) Erlotinib induces the human non-small-cell lung cancer cells apoptosis via activating ROS-dependent JNK pathways. Cancer Med 5(11):3166–3175

Shan L, Wang Z, Guo L, et al (2015) Concurrence of EGFR amplification and sensitizing mutations indicate a better survival benefit from EGFR-TKI therapy in lung adenocarcinoma patients. Lung Cancer 89(3):337–342

Sharma A, Boise LH, Shanmugam M (2019) Cancer metabolism and the evasion of apoptotic cell death. Cancers (Basel) 11(8):1144

Sherr CJ, McCormick F (2002) The RB and p53 pathways in cancer. Cancer Cell 2(2):103–112

Siegel RL, Miller KD, Jemal A (2020) Cancer statistics, 2020. CA Cancer J Clin 70(1):7–30

Sigismund S, Avanzato D, Lanzetti L (2018) Emerging functions of the EGFR in cancer. Mol Oncol 12(1):3–20

Skoulidis F, Heymach JV (2019) Co-occurring genomic alterations in non-small-cell lung cancer biology and therapy. Nat Rev Cancer 19(9):495–509

Sosa Iglesias V, Giuranno L, Dubois LJ, et al (2018) Drug resistance in non-small cell lung cancer: A potential for NOTCH targeting? Front Oncol 8:267

Stjernström A, Karlsson C, Fernandez OJ, et al (2014) Alterations of INPP4B, PIK3CA and pakt of the PI3K pathway are associated with squamous cell carcinoma of the lung. Cancer Med 3(2):337–348

Stretton C, Hoffmann TM, Munson MJ, et al (2015) GSK3-mediated raptor phosphorylation supports amino-acid-dependent mTORC1-directed signalling. Biochem J 470(2):207–221

Sugio K, Uramoto H, Onitsuka T, et al (2009) Prospective phase II study of gefitinib in non-small cell lung cancer with epidermal growth factor receptor gene mutations. Lung Cancer 64(3):314–318

Swaisland HC, Smith RP, Laight A, et al (2005) Single-dose clinical pharmacokinetic studies of gefitinib. Clin Pharmacokinet 44(11):1165–1177

Swanton C (2012) Intratumor heterogeneity: evolution through space and time. Cancer Res 72(19):4875–4882

Syed V (2016) TGF-$\beta$ signaling in cancer. J Cell Biochem 117(6):1279–1287

Tang W, Li X, Xie X, et al (2019) EGFR inhibitors as adjuvant therapy for resected non-small cell lung cancer harboring EGFR mutations. Lung Cancer 136:6–14

Tellez-Gabriel M, Ory B, Lamoureux F, et al (2016) Tumour heterogeneity: The key advantages of single-cell analysis. Int J Mol Sci 17(12):2142

The Cancer Genome Atlas Research Network (2014) Comprehensive molecular profiling of lung adenocarcinoma. Nature 511(7511):543–550

Tomas A, Futter CE, Eden ER (2014) EGF receptor trafficking: consequences for signaling and cancer. Trends Cell Biol 24(1):26–34

Tsubata Y, Hayashi M, Tanino R, et al (2017) Evaluation of the heterogeneous tissue distribution of erlotinib in lung cancer using matrix-assisted laser desorption ionization mass spectrometry imaging. Sci Rep 7(1):12,622

Tubbs A, Nussenzweig A (2017) Endogenous DNA damage as a source of genomic instability in cancer. Cell 168(4):644–656

Turner NC, Reis-Filho JS (2012) Genetic heterogeneity and cancer drug resistance. Lancet Oncol 13(4):e178–85

Umeguchi H, Sueoka-Aragane N, Kobayashi N, et al (2015) Usefulness of plasma HGF level for monitoring acquired resistance to EGFR tyrosine kinase inhibitors in non-small cell lung cancer. Oncol Rep 33(1):391–396

Vallee A, Sagan C, Le Loupp AG, et al (2013) Detection of EGFR gene mutations in non-small cell lung cancer: lessons from a single-institution routine analysis of 1,403 tumor samples. Int J Oncol 43(4):1045–1051

Van Der Steen N, Giovannetti E, Carbone D, et al (2018) Resistance to epidermal growth factor receptor inhibition in non-small cell lung cancer. Canc Drug Resist

Vasconcelos PENS, Gergis C, Viray H, et al (2020) EGFR-A763-Y764insFQEA is a unique exon 20 insertion mutation that displays sensitivity to approved and in-development lung cancer EGFR tyrosine kinase inhibitors. JTO Clin Res Rep 1(3):100,051

Velazquez AI, McCoach CE (2020) Tumor evolution in epidermal growth factor receptor mutated non-small cell lung cancer. J Thorac Dis 12(5):2896–2909

Villaruz LC, Socinski MA (2013) The clinical viewpoint: definitions, limitations of RECIST, practical considerations of measurement. Clin Cancer Res 19(10):2629–2636

Vinay DS, Ryan EP, Pawelec G, et al (2015) Immune evasion in cancer: Mechanistic basis and therapeutic strategies. Semin Cancer Biol 35 Suppl:S185–S198

Vyse S, Huang PH (2019) Targeting EGFR exon 20 insertion mutations in non-small cell lung cancer. Signal Transduct Target Ther 4(1):5

Wang C, Tang Z, Zhao Y, et al (2014) Three-dimensional in vitro cancer models: a short review. Biofabrication 6(2):022,001

Wang S, Zhou Q, Gallo JM (2009) Demonstration of the equivalent pharmacokinetic/pharmacodynamic dosing strategy in a multiple-dose study of gefitinib. Mol Cancer Ther 8(6):1438–1447

Waters CE, Saldivar JC, Hosseini SA, et al (2014) The FHIT gene product: tumor suppressor and genome "caretaker". Cell Mol Life Sci 71(23):4577–4587

Wee P, Wang Z (2017) Epidermal growth factor receptor cell proliferation signaling pathways. Cancers (Basel) 9(5):52

Weinberg RA (2013) The biology of cancer, 2nd edn. Garland Science, London, England

Williams MJ, Sottoriva A, Graham TA (2019) Measuring clonal evolution in cancer with genomics. Annu Rev Genomics Hum Genet 20(1):309–329

Wilson KJ, Mill C, Lambert S, et al (2012) EGFR ligands exhibit functional differences in models of paracrine and autocrine signaling. Growth Factors 30(2):107–116

Wu JY, Wu SG, Yang CH, et al (2008) Lung cancer with epidermal growth factor receptor exon 20 mutations is associated with poor gefitinib treatment response. Clin Cancer Res 14(15):4877–4882

Xie J, Wang X, Proud CG (2016) mTOR inhibitors in cancer therapy. F1000Res 5:2078

Yalamanchili N, Kriete A, Alfego D, et al (2016) Distinct cell stress responses induced by ATP restriction in quiescent human fibroblasts. Front Genet 7:171

Yamamoto H, Shigematsu H, Nomura M, et al (2008) PIK3CA mutations and copy number gains in human lung cancers. Cancer Res 68(17):6913–6921

Yang CH, Yu CJ, Shih JY, et al (2008) Specific EGFR mutations predict treatment outcome of stage IIIB/IV patients with chemotherapy-naive non-small-cell lung cancer receiving first-line gefitinib monotherapy. J Clin Oncol 26(16):2745–2753

Yang F, Chen H, Xiang J, et al (2010) Relationship between tumor size and disease stage in non-small cell lung cancer. BMC Cancer 10(1):474

Yang HM (2012) Mathematical modeling of solid cancer growth with angiogenesis. Theor Biol Med Model 9(1):2

Yang JJ, Zhou Q, Yan HH, et al (2017) A phase III randomised controlled trial of erlotinib vs gefitinib in advanced non-small cell lung cancer with EGFR mutations. Br J Cancer 116(5):568–574

Yarden Y, Pines G (2012) The ERBB network: at last, cancer therapy meets systems biology. Nat Rev Cancer 12(8):553–563

Yarden Y, Sliwkowski MX (2001) Untangling the ErbB signalling network. Nat Rev Mol Cell Biol 2(2):127–137

Yasuda H, Kobayashi S, Costa DB (2012) EGFR exon 20 insertion mutations in non-small-cell lung cancer: preclinical data and clinical implications. Lancet Oncol 13(1):e23–31

Yasuda H, Park E, Yun CH, et al (2013) Structural, biochemical, and clinical characterization of epidermal growth factor receptor (EGFR) exon 20 insertion mutations in lung cancer. Sci Transl Med 5(216):216ra177

Yoshimura A, Yamada T, Okura N, et al (2018) The impact of the tumor shrinkage by initial

EGFR inhibitors according to the detection of EGFR-T790M mutation in patients with non-small cell lung cancer harboring EGFR mutations. BMC Cancer 18(1):1241

You W, Henneberg M (2018) Cancer incidence increasing globally: The role of relaxed natural selection. Evol Appl 11(2):140–152

Yuan M, Huang LL, Chen JH, et al (2019) The emerging treatment landscape of targeted therapy in non-small-cell lung cancer. Signal Transduct Target Ther 4(1):61

Yun CH, Boggon TJ, Li Y, et al (2007) Structures of lung cancer-derived EGFR mutants and inhibitor complexes: mechanism of activation and insights into differential inhibitor sensitivity. Cancer Cell 11(3):217–227

Zegers CML, van Elmpt W, Reymen B, et al (2014) In vivo quantification of hypoxic and metabolic status of NSCLC tumors using [18F]HX4 and [18F]FDG-PET/CT imaging. Clin Cancer Res 20(24):6389–6397

Zhai X, Ward RA, Doig P, et al (2020) Insight into the therapeutic selectivity of the irreversible EGFR tyrosine kinase inhibitor osimertinib through enzyme kinetic studies. Biochemistry 59(14):1428–1441

Zhan T, Rindtorff N, Boutros M (2017) Wnt signaling in cancer. Oncogene 36(11):1461–1473

Zhang H (2016) Osimertinib making a breakthrough in lung cancer targeted therapy. Onco Targets Ther 9:5489–5493

Zhang J, Gold KA, Lin HY, et al (2015) Relationship between tumor size and survival in non-small-cell lung cancer (NSCLC): an analysis of the surveillance, epidemiology, and end results (SEER) registry. J Thorac Oncol 10(4):682–690

Zheng X, Sweidan M (2018) A mathematical model of angiogenesis and tumor growth: analysis and application in anti-angiogenesis therapy. J Math Biol 77(5):1589–1622
